# Fine Tuning Inflammation at the Front Door: Macrophage Complement Receptor 3-mediates Phagocytosis and Immune Suppression for *Francisella tularensis*

**DOI:** 10.1371/journal.ppat.1003114

**Published:** 2013-01-24

**Authors:** Shipan Dai, Murugesan V. S. Rajaram, Heather M. Curry, Rachel Leander, Larry S. Schlesinger

**Affiliations:** 1 Center for Microbial Interface Biology, Department of Microbial Infection and Immunity, The Ohio State University, Columbus, Ohio, United States of America; 2 Mathematical Biosciences Institute, The Ohio State University, Columbus, Ohio, United States of America; Stanford University School of Medicine, United States of America

## Abstract

Complement receptor 3 (CR3, CD11b/CD18) is a major macrophage phagocytic receptor. The biochemical pathways through which CR3 regulates immunologic responses have not been fully characterized. *Francisella tularensis* is a remarkably infectious, facultative intracellular pathogen of macrophages that causes tularemia. Early evasion of the host immune response contributes to the virulence of *F. tularensis* and CR3 is an important receptor for its phagocytosis. Here we confirm that efficient attachment and uptake of the highly virulent Type A *F. tularensis* spp. *tularensis* strain Schu S4 by human monocyte-derived macrophages (hMDMs) requires complement C3 opsonization and CR3. However, despite a>40-fold increase in uptake following C3 opsonization, Schu S4 induces limited pro-inflammatory cytokine production compared with non-opsonized Schu S4 and the low virulent *F. novicida*. This suggests that engagement of CR3 by opsonized Schu S4 contributes specifically to the immune suppression during and shortly following phagocytosis which we demonstrate by CD11b siRNA knockdown in hMDMs. This immune suppression is concomitant with early inhibition of ERK1/2, p38 MAPK and NF-κB activation. Furthermore, TLR2 siRNA knockdown shows that pro-inflammatory cytokine production and MAPK activation in response to non-opsonized Schu S4 depends on TLR2 signaling providing evidence that CR3-TLR2 crosstalk mediates immune suppression for opsonized Schu S4. Deletion of the CD11b cytoplasmic tail reverses the CR3-mediated decrease in ERK and p38 activation during opsonized Schu-S4 infection. The CR3-mediated signaling pathway involved in this immune suppression includes Lyn kinase and Akt activation, and increased MKP-1, which limits TLR2-mediated pro-inflammatory responses. These data indicate that while the highly virulent *F. tularensis* uses CR3 for efficient uptake, optimal engagement of this receptor down-regulates TLR2-dependent pro-inflammatory responses by inhibiting MAPK activation through outside-in signaling. CR3-linked immune suppression is an important mechanism involved in the pathogenesis of *F. tularensis* infection.

## Author Summary

The highly virulent *Francisella tularensis* can cause respiratory disease in humans with less than 10 bacteria. *In vivo*, it replicates mainly within macrophages. Evasion and/or suppression of the host protective immune response is essential to *Francisella's* virulence in mammals. However, the detailed molecular mechanisms for this immune suppression are not clear. Here we demonstrate that this pathogen manipulates the crosstalk between two important receptor-mediated pathways during entry in human macrophages: CR3-mediated phagocytosis and TLR2-mediated pro-inflammatory responses. By optimally engaging CR3, *Francisella* not only gains efficient access to macrophages but also dampens TLR2-mediated immune responses, leading to a relatively “silent” entry that allows it to replicate intracellularly to high numbers early on. Thus, we have identified an important mechanism that is critical for the success of *F. tularensis* as a human pathogen during primary infection.

## Introduction

*Francisella tularensis* is a remarkably infectious facultative intracellular pathogen that causes the zoonotic disease tularemia [1], [2]. *F. tularensis* can be divided into several subspecies, including *tularensis*, *holarctica* and *mediasiatica* [3], [4], with *tularensis* being the most virulent subspecies that can cause disease in humans through the respiratory route with <10 CFUs. *F*. *novicida* is normally considered to be the fourth subspecies of *F. tularensis*; however, recent genome-wide polymorphism analysis indicates that it is an independent species [5]. *F. novicida* rarely causes disease in humans but is virulent in mice, manifesting a disease that is similar to tularemia in humans, and has been widely used in the mouse model of tularemia. Because *F. tularensis* can be easily disseminated, results in a high mortality rate among untreated pulmonary cases, and has the potential to cause public panic, it is given the highest priority classification by the Centers for Disease Control and Prevention (CDC) as a Category A select agent and is a potential bioweapon [1].

Macrophages are the first line of defense against invading microorganisms. However, as is the case for many other intracellular pathogens, *F. tularensis* is able to avoid and/or suppress macrophage host defense mechanisms to enable survival and intracellular replication following phagocytosis. *F. tularensis* infects primarily alveolar macrophages in pneumonic tularemia [6]. Macrophages combat *F. tularensis* infection by generating TLR2-dependent pro-inflammatory cytokines such as TNF-α and IL-1β [7]–[12]. However, this cytokine response is largely muted at the early stage of *F. tularensis* infection. In fact, several studies have indicated that *F. tularensis* infection leads to broad immune suppression in infected cells [13]–[16]. Compared with the low virulent *F. novicida*, infection of human phagocytes with the highly virulent Type A *F. tularensis* subsp. *tularensis* strain Schu S4 leads to significantly reduced pro-inflammatory cytokine production [17], [18]. This immune suppression is believed to be critical for the success of *F. tularensis* as a human pathogen, allowing for its replication and dissemination within the host early on. However, the molecular mechanisms involved in the early *F. tularensis*-induced immune suppression are not completely understood.

Multiple receptors, such as complement receptors, Fcγ receptors, mannose receptor, scavenger receptor and surface nucleolin have all been implicated in *F. tularensis* phagocytosis [19]–[24]. Complement C3 deposition on the bacterial surface, and complement receptors, specifically complement receptor 3 (CR3) on the macrophage surface play important roles in the uptake of the highly virulent *F. tularensis* subsp. *tularensis* by human macrophages [20], [21], [25]. CR3 (CD11b/CD18, α_M_β_2_) belongs to the β_2_-integrin family. Complement receptors, particularly CR3, have long been postulated to allow for safe passage for intracellular pathogens [26]–[28]. CR3's function is dependent on the activation of outside-in and inside-out two way signals [29]. There is increasing evidence for signaling crosstalk between complement receptors and TLRs [30]–[32]. For example, TLR2 is able to trans-activate CR3 through inside-out signaling including the activation of Rac1, PI3K and cytohesin-1 [33], [34]. β_2_-integrin signaling can also negatively regulate TLR responses [32], [35]. Specifically, CR3 can inhibit TLR4 signaling by promoting the degradation of MyD88 and TRIF [36]. In addition, engagement of CR3 has been shown to down-regulate IL-12 production [37] and avoid initiation of the oxidative burst in macrophages following phagocytosis of apoptotic cells [38], [39]. A few pathogens such as *Porphyromonas gingivalis* [33], *Mycobacterium bovis BCG* [34] and *Bacillus anthracis* spores [40] can activate CR3 through inside-out signaling via TLR2 to facilitate bacterial uptake. However, to the best of our knowledge, nothing is known about how the engagement of CR3 by a pathogen (i.e. outside-in signaling) can mediate regulation of TLR signaling pathways.

Here we hypothesized that the initial interaction of *F. tularensis* with human macrophage receptors will greatly impact the generation of early signals and consequent biological responses by the macrophage. We show that C3-opsonized virulent *F. tularensis* subsp. *tularensis* utilizes CR3 to gain access into host macrophages while at the same time inhibits TLR2-mediated host immune responses, resulting in a relatively “silent” mode of entry followed by robust intracellular replication. We also report the key signaling pathways involved. Thus, we provide the first evidence for linkage between a key phagocytic receptor for *F. tularensis* and post-phagocytic signaling events important in immunosuppression. The involvement of complement receptors in immune suppression may be an important mechanism for *F. tularensis* pathogenesis, and the pathways involved are likely to be broadly applicable to other intracellular pathogens that use CR3-mediated entry.

## Results

### Serum opsonization, specifically C3 deposition, is critical for *F. tularensis* subsp. *tularensis* Schu S4 phagocytosis by human macrophages

**Figure 1:**
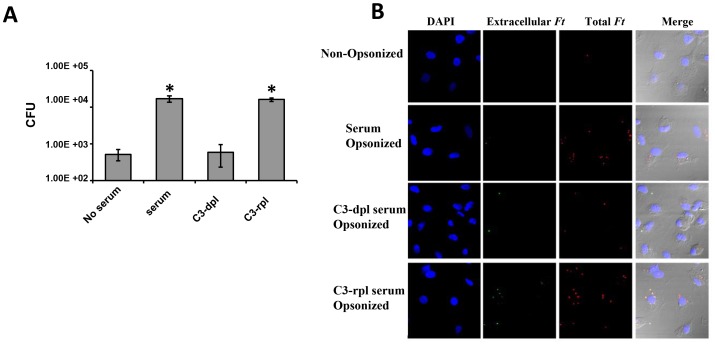
C3 is required for *Ft* Schu S4 uptake by human macrophages. hMDMs were incubated with Schu S4 for 1 hr at an MOI of 50∶1 in the presence or absence of 10% autologous serum, C3-depleted serum (C3-dpl) or C3-repleted serum (C3-rpl). (A) Extracellular bacteria were killed by gentamycin treatment for 30 min at 37°C. hMDMs were then lysed and intracellular bacteria were enumerated by CFUs recovered. Data are representative of three independent experiments. The data were analyzed by 1-Way ANOVA and Tukey's Multiple Comparison Test (*p<0.05 serum vs. no serum and C3-rpl vs. C3-dpl). (B) hMDMs were fixed after 1 hr of infection and followed by inside/outside differential staining to differentiate intracellular (red) and extracellular (yellow or green) bacteria as described in Material and Methods. Images shown are representatives of three independent experiments. In the experiment shown, quantification of the total number of Sch S4 per cell was as follows: no serum = 0.35; serum = 4.04; C3-dpl. = 0.269; C3-rpl serum = 3.757 (150 cells per experimental group).

Serum components, specifically complement C3, and CR3 play a major role in efficient macrophage uptake of *F. tularensis* Live Vaccine Strain (LVS) and a clinical isolation (RCI) of subsp. *tularensis* [21]. We first set out to confirm the role of C3 and C3 receptors in Schu S4 phagocytosis by human monocyte-derived macrophages (hMDMs). We infected hMDMs with Schu S4 in the presence or absence of serum, or in C3-depleted or C3-repleted serum. As shown in Fig. 1, in the absence of serum, Schu S4 uptake by hMDMs was very limited. In contrast, in the presence of serum, the uptake was significantly increased ∼40 fold confirming that serum opsonization is critical for efficient phagocytosis of Schu S4 by human macrophages. Moreover, when we used C3-depleted serum the uptake of Schu S4 was limited, equivalent to that seen in the absence of serum. Repletion of C3-depleted serum with C3 led to a significant increase in uptake akin to that seen following serum opsonization. This result provides further evidence that serum components, especially C3 deposited on bacterial surface, are critical for efficient phagocytosis of Schu S4 by human macrophages.

### C3 opsonization is associated with immune suppression during Schu S4 phagocytosis by macrophages

**Figure 2:**
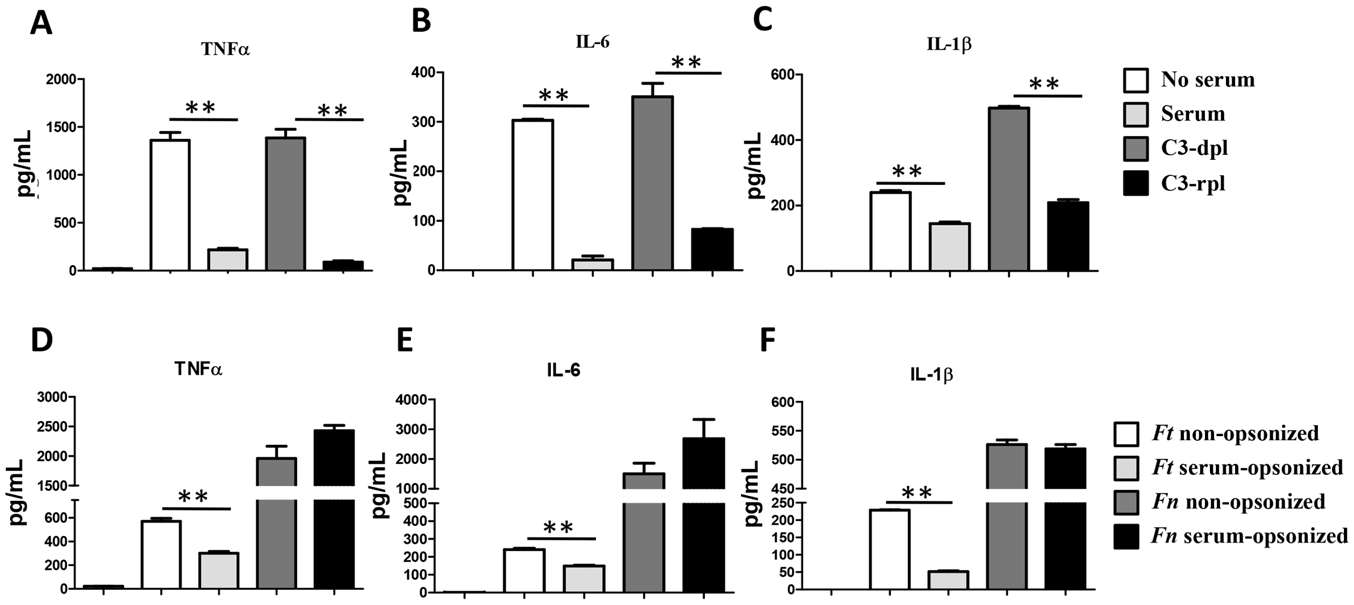
Serum components, specifically C3, have an inhibitory effect on pro-inflammatory cytokine production upon Schu S4 infection of human macrophages. (A–C) hMDMs were infected with *Ft* Schu S4 at an MOI of 50∶1 in RHH or RHS with 10% autologous serum, C3-depleted serum (C3d) or C3-repleted serum (C3r) for 1 h; or (D–F) hMDMs were infected with *Ft* Schu S4 (Ft) or *F. novicida* (Fn) at an MOI of 50∶1 in RHH or RHS with 10% autologous serum. Extracellular bacteria were killed with 50 µg/ml gentamycin at 37°C for 30 min. Media were replenished and cell-free culture supernatants were collected at 16 hrs post infection. TNFα, IL-6 and IL-1β concentrations were measured by ELISAs. Uninfected resting cells (R) were included as a control. Data are representative of at least 3 independent experiments. The data were analyzed by 1-Way ANOVA and Tukey's Multiple Comparison Test. ** p<0.005.

In order to examine whether the initial interaction between *F. tularensis* and macrophages impacts host cell immune responses, we measured the levels of pro-inflammatory cytokines, *i.e.* TNFα, IL-6 and IL-1β in the culture supernatant of hMDMs infected with Schu S4 in the absence or presence of fresh autologous serum. Surprisingly, despite the fact that there was a dramatic increase in bacterial uptake in the presence of serum, there was very limited pro-inflammatory cytokine production suggesting that serum opsonization has an immunosuppressive effect during Schu S4 phagocytosis. This effect is mainly contributed by C3 since incubation in C3-depleted serum led to relatively high levels of cytokine production comparable to those observed in the absence of serum, while the cytokine levels were decreased when C3 was repleted (Fig. 2A, B and C). Recent studies have shown that the low virulent *F. novicida* induces high pro-inflammatory responses in human phagocytic cells [17], [41]. In order to exam whether serum incubation has an immunosuppressive effect during the phagocytosis of human macrophages by *F. novicida*, we performed assays comparing *F. novicida* and Schu S4 in the presence and absence of serum. Consistent with previous studies [17], [18], phagocytosis of *F. novicida* by hMDMs was accompanied by much higher pro-inflammatory cytokine production than that of Schu S4 (Fig. 2 D, E and F). In addition, serum did not have a significant immune suppressive effect, indicating that C3 opsonin-mediated immune suppression is specific to the highly virulent Schu S4.

Recent studies have indicated that host adaptation can affect the nature of *Francisella* surface carbohydrates, which can in turn affect the recognition by complement components and TLRs [42]. To examine the effect of host adaptation on the immune suppression observed here, we used Schu S4 that had been passaged through THP-1 cells (Ft-T). As shown in Fig. S1, although host adapted Ft-T induced slightly less pro-inflammatory cytokine production compared with Ft that had not been passaged, serum-opsonization still significantly inhibited pro-inflammatory responses with Ft-T. This result provides further support that the inhibition mechanism observed here does not depend on host adaptation. Thus, the following experiments were carried out with Ft Schu S4 only.

### C3 opsonization inhibits MAPK activation during phagocytosis of Schu S4 by human macrophages

**Figure 3:**
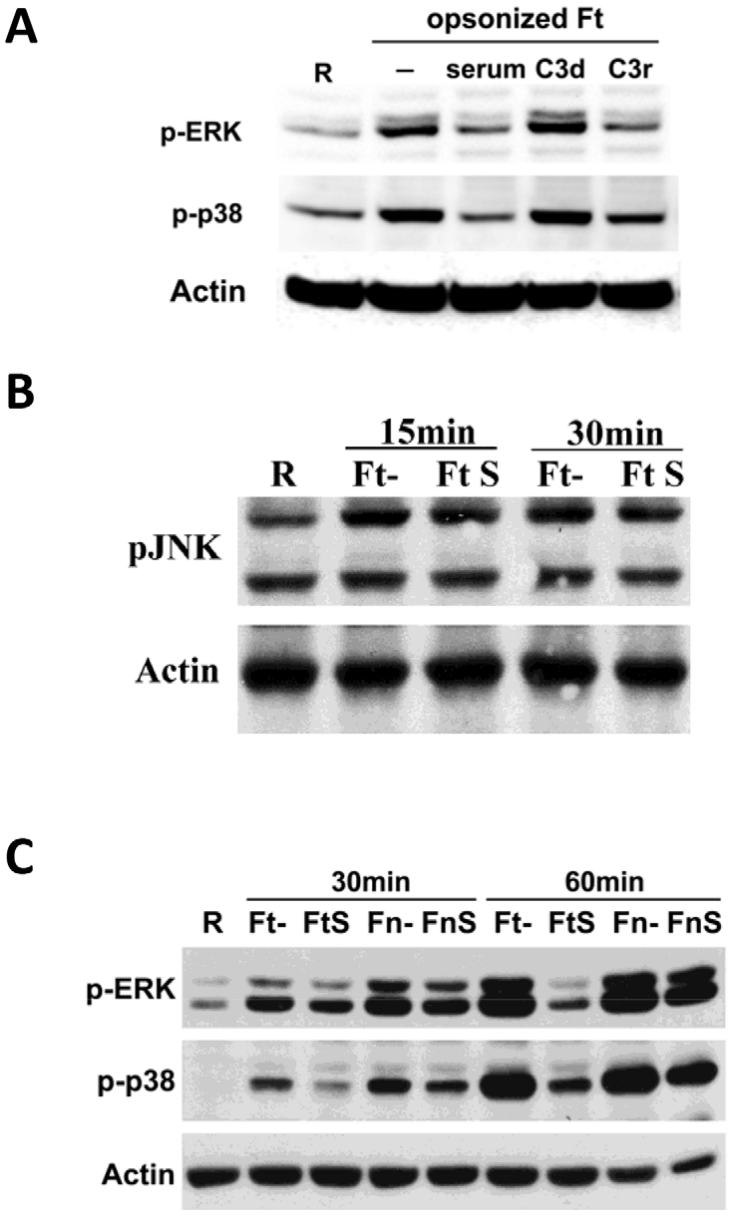
Serum components, specifically C3 opsonization, inhibit ERK1/2 and p38 activation during Schu S4 phagocytosis by macrophages. (A) Schu S4 was either non-opsonized, or pre-opsonized with 10% autologous serum, C3-depleted serum (C3d) or C3-repleted serum (C3r), and then used to infect hMDM monolayers at an MOI of 50∶1 in RHH in the absence of serum. Infection was synchronized by centrifuging at 250×g for 10 min at 4°C, and incubated at 37°C for 30 min. (B) Schu S4 was either non-opsonized, or pre-opsonized with 10% autologous serum, and then used to infect hMDM monolayers at an MOI of 50∶1 in RHH in the absence of serum. Infection was synchronized by centrifuging at 250×g for 10 min at 4°C, and incubated at 37°C for 15 or 30 min. (C) Schu S4 (Ft) or *F. novicida* (Fn) were either non-opsonized or serum pre-opsonized, and then used to infect hMDMs as in (A) for 30 or 60 min. MDMs lysates were subjected to Western Blot. Uninfected resting cells (R) were included as control. Data are representative of 3 independent experiments.

*F. tularensis* LPS interacts poorly with TLR4 [43]. Instead, host inflammatory responses to *F. tularensis* are mediated primarily by TLR2 which leads to the activation of MAPK and NF-κB signaling pathways, and pro-inflammatory cytokine production [44]–[49]. In order to explore the underlying mechanism for the suppressed immune responses seen in the presence of serum and C3 opsonization, we examined the activation of MAPKs during Schu S4 synchronized phagocytosis. Schu S4 was either non-opsonized or pre-opsonized with fresh autologous serum, C3-depleted or C3-repleted serum, and then incubated with hMDMs in the absence of serum. As shown in Fig. 3A, consistent with our observations of pro-inflammatory cytokine production, activation of MAPKs (i.e. ERK1/2 and p38) was inhibited during phagocytosis of serum-opsonized or C3-repleted serum-opsonized Schu S4, whereas there was robust activation of ERK1/2 and p38 in the absence of either serum or C3. This inhibition on MAPK activation was only observed for ERK1/2 and p38, but not for JNK (Fig. 3B) In order to confirm the role of complement in the immune suppression observed, we also infected hMDMs with Schu S4 pre-opsonized with heat-inactivated autologous serum. Phagocytosis of these bacteria was accompanied by levels of ERK1/2 and p38 activation equivalent to those seen with non-opsonized Schu S4 (data not shown). Together, these data indicate that the immune suppression during C3-opsonized Schu S4 phagocytosis is mediated, at least in part, by reduced ERK1/2 and p38 activation.

Since we observed a much greater pro-inflammatory response during *F. novicida* phagocytosis than during Schu S4 phagocytosis (which was not affected by serum or C3), we also included *F. novicida* in parallel with Schu S4 in our experiments to assess MAPK activation. As expected, phagocytosis of *F. novicida* was accompanied by high levels of ERK1/2 and p38 activation both in the presence and absence of serum, especially at the later time point (Fig. 3C) and was much less affected by serum opsonization. This indicates that C3-mediated MAPK inhibition is relatively specific to the highly virulent Schu S4.

### C3 opsonization inhibits NF-κB p65 activation and translocation during phagocytosis of Schu S4 by human macrophages

**Figure 4:**
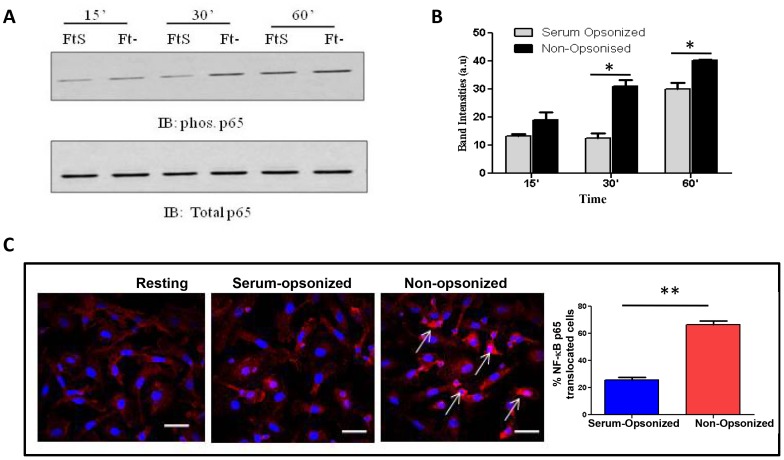
Serum opsonization of Schu S4 leads to inhibition of NF-κB p65 phosphorylation and translocation into nucleus during phagocytosis by macrophages. (A) hMDMs were infected with pre-opsonized (10% autologous serum) or non-opsonized Schu S4 at an MOI of 50∶1 in RHH. Infection was synchronized by centrifuging at 250×g for 10 min at 4°C, and incubated at 37°C for different time points (15′, 30′ and 60′). MDM lysates were subjected to Western Blot using Phospho NF-κB p65 or total p65 primary antibodies. (B) Cumulative data of band intensities from n = 2 (* p<0.05). (C) hMDMs were fixed after 1 hr of infection with pre-opsonized or non-opsonized Schu S4 at an MOI of 50∶1 and NF-κB p65 translocation was examined by immunofluorescence microscopy with rabbit NF-κB p65 antibody followed by anti-rabbit AF 594 antibody. Nuclei were stained with DAPI. NF-κB p65 translocation was scored using a total of 300 cells per experiment. The graph shown represents cumulative data from two independent experiments. The data were analyzed by a two-tailed Student t-test (** p<0.005, Student t-test).

Phosphorylation of MAPKs leads to activation of many transcription factors, including NF-κB, that are important in inflammatory cytokine production. Since we observed significantly decreased activation of ERK 1/2 and p38 during serum-opsonized Schu S4 infection, we examined whether this decreased activation leads to a reduction in downstream NF-κB activation. We infected hMDMs with non-opsonized or serum-opsonized Schu S4 for different time points and analyzed NF-κB p65 activation (phosphorylation) by Western blot. As predicted, phagocytosis of serum-opsonized Schu S4 led to significantly reduced phosphorylation of the NF-κB p65 subunit (Fig. 4A and B). Activation of the NF-κB p65 subunit leads to translocation of p65 into the nucleus where it binds to the promoter region of various genes to induce their expression. We measured NF-κB p65 translocation into the nucleus by immunofluorescence microscopy (Fig. 4C). Our results show that infection with serum-opsonized Sch S4 is accompanied by significantly reduced p65 translocation compared to that of non-opsonized bacteria. These data provide strong evidence that C3 opsonization of bacteria leads to early suppression of the host cell immune response through inhibition of MAPKs and downstream NF-κB activation.

### C3 opsonin-mediated immune suppression during phagocytosis is not due to a difference in the kinetics of uptake of Schu S4 or to bacterial factors

**Figure 5:**
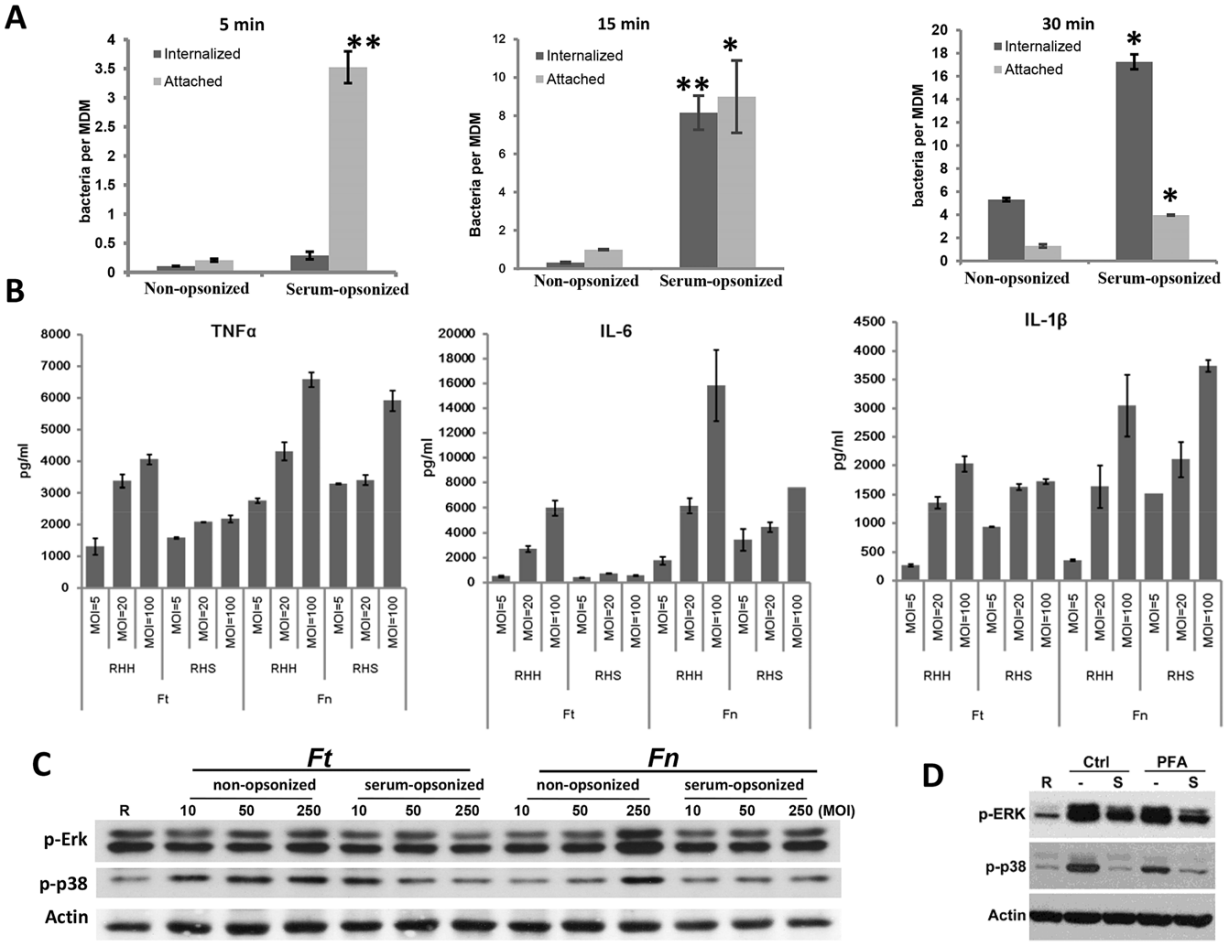
C3-mediated immune suppression is not due to a difference in the kinetics of Schu S4 phagocytosis. (A) In the presence of serum, there were more *Ft* Schu S4 attachment and phagocytosis. hMDMs were infected with non-opsonized or serum pre-opsonized *Ft* Schu S4 at an MOI of 50∶1. Infections were synchronized by centrifugation at 4°C for 10 min at 250×g. At 5, 15 and 30 min post infection samples were washed extensively, fixed with 2% paraformaldehyde (PFA), and followed with inside/outside differential staining. The numbers of attached (extracellular) and phagocytosed (intracellular) bacteria were counted under an epi-fluorescence microscope. At least 300 cells were counted for every sample. Data are representative of 3 independent experiments performed in triplicate (mean ± SD). Non-opsonized vs. serum-opsonized, * p<0.05, ** p<0.005 Student t-test. (B) hMDMs were infected with Schu S4 (*Ft*)or *F. novicida* (*Fn*) at MOIs of 5, 20 and 100 in the presence (RHS with 10% autologous serum) or absence (RHH) of serum. Cell-free culture supernatants were collected after 16 h and cytokine levels were measured by ELISA. Data are representative of 3 independent experiments performed in triplicate (mean ± SD). (C) hMDMs were infected with non-opsonized or serum pre-opsonized Schu S4 (*Ft*) or *F. novicida* (*Fn*) at MOIs of 10, 50 or 250 for 30 min. Cell lysates were subjected to Western blot analysis. Data are representative for 3 independent experiments. (D) Schu S4 bacteria were either killed with paraformaldehyde or incubated with PBS (control) for 10 min at room temperature. hMDMs were then infected with non-opsonized or serum pre-opsonized live or PFA-killed Schu S4 at MOI of 50∶1 for 30 min. Cell lysates were subjected to Western blot analysis. Data are representative of 3 independent experiments.

One possible mechanism for the enhanced pro-inflammatory cytokine response seen during phagocytosis of non-opsonized Schu S4 is that limited CR3 engagement slows phagocytosis (through CR3 or potentially other receptors) allowing for a proportionately increased number of attached bacteria to engage TLR2 for a longer period of time. In order to test for this possibility, we synchronized phagocytosis of non-opsonized or serum-opsonized Schu S4 as in the MAPK activation assay, and fixed the samples at different time points. We used a differential immunofluorescence staining protocol [50] to quantify the attachment and uptake of Schu S4 by hMDMs (Fig. 5A). At all time points examined (5, 15 and 30 min) there were much greater numbers of serum-opsonized Schu S4 both attached to and taken up by hMDMs compared with non-opsonized bacteria, indicating that a difference in the kinetics of phagocytosis was not the reason for the observed immune suppression. The fact that *F. tularensis* can also signal through TLR2 from within phagosomes following phagocytosis [51] also argues against the hypothesis that in the case of non-opsonized bacteria, prolonged cell surface binding results in enhanced cytokine production.

Bacterial virulence factors, e.g. RipA, represent an alternate mechanism of immune suppression by C3-opsonized bacteria [52]. In this scenario, we expect cytokine production to decline as bacterial loads increase because the concentration of virulence factors produced from viable bacteria within a macrophage is directly proportional to its bacterial load. In order to test this hypothesis, we infected hMDMs with non-opsonized or serum pre-opsonized Schu S4. In both cases we achieved an incremental increase in bacterial uptake by increasing the MOI. However, instead of resulting in increased immune suppression, increased uptake resulted in a stepwise increase in pro-inflammatory cytokine production (Fig. 5B) and MAPK activation (Fig. 5C). This effect was more pronounced in the case of non-opsonized bacteria [i.e., A 20 fold increase in MOI resulted in a 3 to 12 fold increase in cytokine production for non-opsonized bacteria, and only a 30% to 70% increase in cytokine production for opsonized bacteria (Fig. 5B)]. These results indicate that early immune suppression by Schu S4 during phagocytosis is not primarily mediated by virulence factors produced from viable bacteria. We carried out experiments with paraformaldehyde (PFA)-killed Schu S4 to further support this claim. In parallel with untreated Schu S4, PFA-killed Schu S4 was either non-opsonized or serum pre-opsonized, and used for infection. As shown in Fig. 5D, PFA-killed serum-opsonized Schu S4 was able to inhibit MAPK activation. Together, these results provide evidence that the immune suppression observed during phagocytosis of C3-opsonized Schu S4 is not due to actively produced bacterial factors. However, these factors are predicted to play an important role during subsequent stages of infection.

### CR3 is the major receptor for Schu S4 phagocytosis by human macrophages

**Figure 6:**
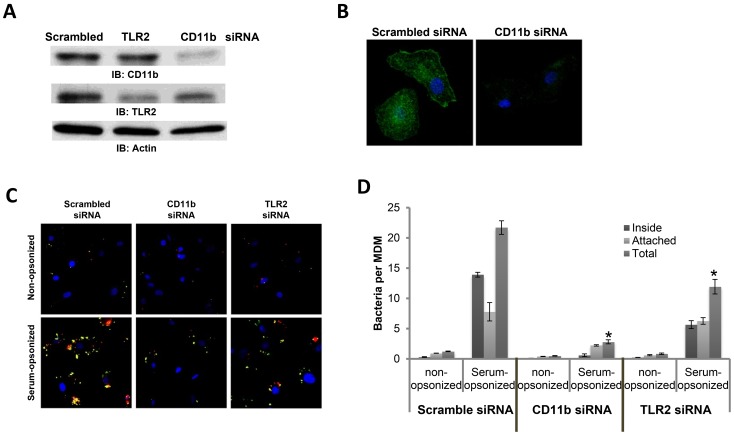
CR3, partially activated by TLR2 inside-out signaling, is critical for Schu S4 phagocytosis by hMDMs. (A) hMDMs were transfected with scrambled siRNA or siRNAs targeting CD11b or TLR2. 48 hrs later, the level of CD11b and TLR2 were examined by Western blot. (B) CD11b knockdown was also examined by immunofluorescence microscopy with mouse CD11b (M1/70) antibody. Nuclei were stained with DAPI. (C) 48 h after siRNA transfection hMDMs were infected with non-opsonized or serum pre-opsonized *Ft* Schu S4 in the absence of serum for 15 min. Infected cells were subjected to differential staining of extracellular (yellow or green) and intracellular (red) bacteria. Representative images are shown from 3 independent experiments. (D) Bacterial uptake was quantified as the number of bacteria that are inside or attached per cell. At least 300 cells were counted for every sample. Data are representative of 3 independent experiments performed in triplicate (mean ± SD). * p<0.05, compared with control siRNA samples (1-Way ANOVA and Tukey's Multiple Comparison Test).

We next examined the role of C3 receptors and the possible crosstalk between C3 receptors and TLR2 in mediating the immune suppression during phagocytosis of C3-opsonized Schu S4. We elected to focus on CR3 (CD11b/CD18) which is the major receptor for C3 opsonins, and has been implicated as the important receptor for Schu S4 phagocytosis by human phagocytes [21]. We transfected hMDMs with scramble siRNA (control) or siRNA targeting CD11b, the latter to knockdown the α chain of the CR3 heterodimer (Fig. 6A and B). We then performed experiments that quantify attachment and uptake of Schu S4 by control and CD11b knockdown macrophages. Compared to control siRNA transfected hMDMs, Schu S4 attachment and uptake were nearly abolished in CD11b knockdown cells, providing strong confirmatory evidence that CR3 is the major receptor for phagocytosis of the highly virulent Schu S4 strain by human macrophages (Fig. 6C and D).

Several studies have indicated crosstalk between complement receptor pathways and TLR pathways, especially for the involvement of TLRs in inside-out activation of CR3 [33], [34], [40]. Therefore, we tested the possible involvement of TLR2 in Schu S4 phagocytosis by hMDMs, since TLR2 is responsible for most of the cell surface TLR-driven host inflammatory responses following *F. tularensis* infection [44], [47]–[49]. We knocked down TLR2 in hMDMs using siRNA as we have described previously [53] and examined Schu S4 attachment and uptake by these cells. Compared with control siRNA transfected cells, there was an approximately 40% reduction in the attachment and uptake of Schu S4 in TLR2 siRNA transfected cells (thus less than that observed with CD11b knockdown cells) at 15 min (Fig. 6C and D), which is consistent with the involvement of TLR2 in *F. tularensis* phagocytosis, possibly through inside-out activation of CR3. Similar data were seen at 5 min and a similar trend was seen at 30 min, although at the later time point the high number of bacteria per cell reduced the accuracy of the counting (Fig. S2). Inside-out signaling is further supported by an experiment showing that the cytoskeletal, actin-binding protein Talin co-localizes with CR3, particularly in macrophages infected with serum pre-opsonized *Ft* (Fig. S3) [54].

### Phagocytosis-associated immune suppression of C3-opsonized *F. tularensis* is mediated by CR3

**Figure 7:**
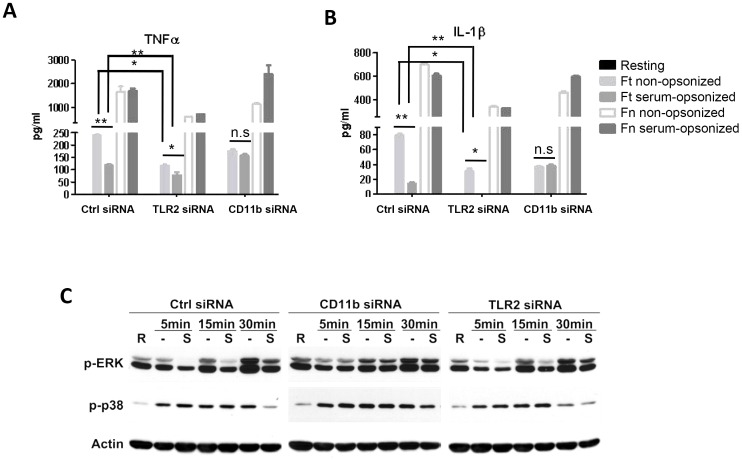
CR3 inhibits while TLR2 activates pro-inflammatory responses in human macrophages after Schu S4 infection. hMDMs were transfected with scrambled siRNA or siRNA targeting CD11b or TLR2. 48 hrs after transfection cells were infected with Schu S4 (*Ft*) or *F. novicida* (*Fn*) in the presence or absence of serum at an MOI of 50∶1. TNFα (A) and IL-1β (B) levels at 16 h post infection in the culture supernatants were measured by ELISAs. Data are representative of 3 independent experiments performed in triplicates. The data were analyzed by 1-Way ANOVA and Tukey's Multiple Comparison Test. * p<0.05, **p<0.005, n.s., not significant. (C) After siRNA transfection hMDMs were infected with non-opsonized or serum pre-opsonized *Ft* Schu S4 at an MOI of 250∶1. Infections were synchronized as described in the materials and methods. Cell lysates were collected at 5, 15 and 30 min post infection and subjected to Western blot analysis using antibodies against phospho-ERK1/2, phospho-p38 and β-actin. Uninfected resting cells (R) were also included as control. Results are representative of at least 3 independent experiments.

In order to test the hypothesis that engagement of C3 receptors enables outside-in crosstalk with the TLR2 pathway to inhibit TLR2-mediated induction of pro-inflammatory responses, we examined cytokine production in CD11b or TLR2 knockdown macrophages following phagocytosis of serum-opsonized versus non-opsonized Schu S4 or *F. novicida*. We observed a significant reduction in pro-inflammatory cytokine levels in TLR2 knockdown cells following non-opsonized or serum-opsonized Schu S4 infection, consistent with a role for TLR2 in mediating host pro-inflammatory immune responses (Fig. 7A). However, even in TLR2 knockdown cells, cytokine production in response to serum-opsonized bacteria remained depressed as compared to non-opsonized bacteria, indicating that the immunosuppressive effects of complement C3 in conjunction with Schu S4 do not depend on TLR2. In contrast, in CD11b knockdown cells, we observed that the selective immune suppression seen with opsonized bacteria was abolished, providing evidence for CR3 in mediating immune suppression following optimal engagement by C3-opsonized Schu S4 (Fig. 7B). As observed before, there was robust pro-inflammatory cytokine production following phagocytosis of *F. novicida* independent of opsonization status.

We next examined MAPK activation upstream of cytokine production following phagocytosis of Schu S4 by control, CD11b or TLR2 knockdown hMDMs. In order to detect and examine the signaling events immediately after receptor ligation, we increased the MOI to 250∶1 and collected samples at 5, 15 and 30 min post infection. As shown in Fig. 7C, in control siRNA transfected cells, both ERK and p38 activation were selectively inhibited upon infection with serum pre-opsonized bacteria compared with non-opsonized bacteria, although maximal inhibition of each occurred at different time points (ERK and p38 activation were significantly lower at 5 min and 30 min post infection, respectively). Similar to the results in Fig. 7A, in TLR2 knockdown cells MAPK activation was reduced and the overall pattern between opsonized and non-opsonized bacteria was maintained; whereas in CD11b knockdown cells, enhanced activation was observed for both ERK1/2 and p38 following infection with serum-opsonized bacteria, now equivalent to the level seen with non-opsonized bacteria. Remarkably this occurred despite the fact that there was minimal phagocytosis by these cells. Together, these results are consistent with a positive role for TLR2 and a negative role for CR3 in MAPK activation upon Schu S4 infection of human macrophages.

### Phagocytosis-associated immune suppression of serum-opsonized Schu S4 is mediated by the CR3 cytoplasmic tail

**Figure 8:**
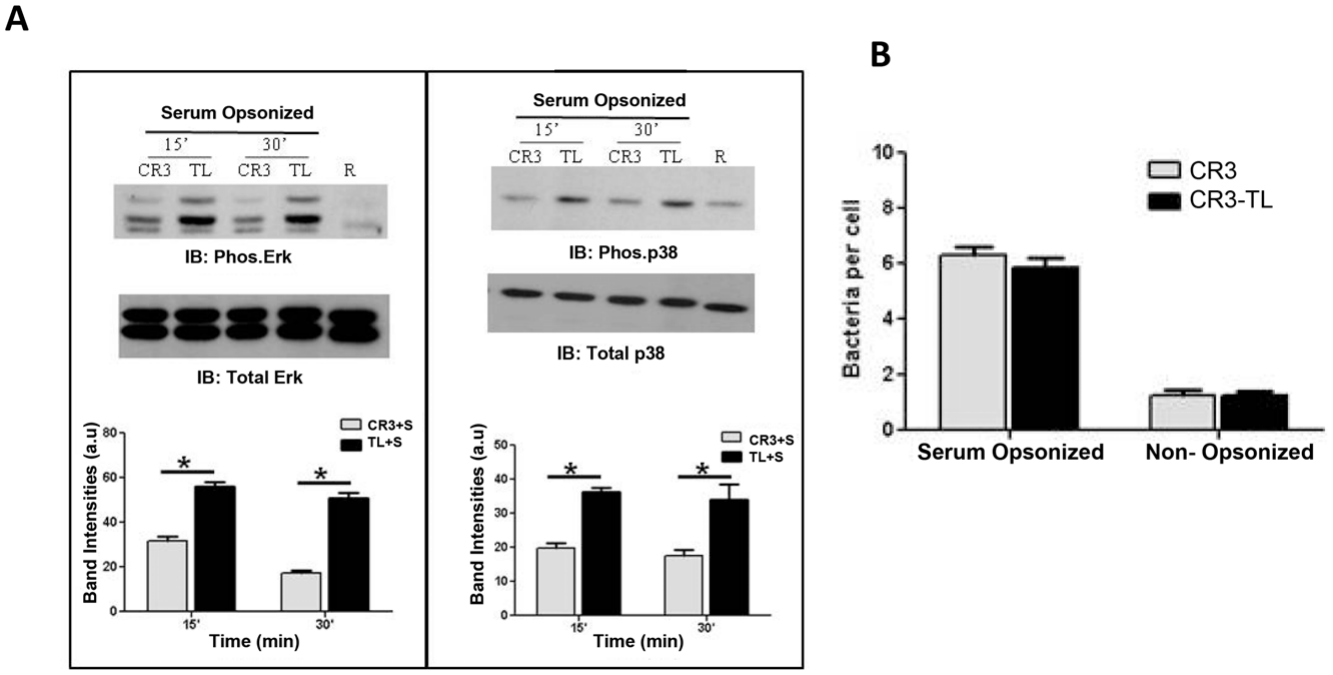
The CR3 cytoplasmic tail is critical for serum opsonin-mediated immune suppression in human macrophages. (A) CHO cells stably expressing full length CR3 or a tail less mutant form of the receptor were transiently transfected with functional TLR2 and subsequently infected with serum pre-opsonized or non-opsonized *Ft* Schu S4 at an MOI of 50∶1 for different time points. Cell lysates were used to examine for ERK, p38 and Akt activation by Western blot using phosphor-specific antibodies. The same membrane was re-probed with total ERK, p38 and Akt as loading controls. The lower panel shows the band intensities measured by Image J software. (B) Cell association was quantified as the number of bacteria per cell by immunofluorescence microscopy using mouse *Ft* LPS antibody followed by Alexa Fluor 488. At least 300 cells were counted for every sample. Data are representative of 2 independent experiments performed in triplicate.

The cytoplasmic tail of CR3 plays an important role in CR3-mediated signaling [55]. To directly assess the role of the CR3 cytoplasmic tail in mediating immune suppression pathway(s) during the phagocytosis of serum-opsonized Schu S4, we used CHO cells stably expressing full length heterodimeric CR3 (CD11b/CD18) or a tail less mutant of CR3 (tail less CD11b along with CD18) and transiently transfected with functional TLR2 [56]. Following incubation of serum-opsonized or non-opsonized bacteria for 15 or 30 min, CHO cell lysates were harvested and analyzed for activation of ERK and p38 activation (phosphorylation) by Western blot. We found that the tail less mutant form of CR3 was not able to inhibit the activation of ERK1/2 and p38 during serum-opsonized Schu-S4 phagocytosis (Fig. 8A). To further validate that CR3 is an important receptor for serum-opsonized Schu S4, we performed a cell association assay with serum-opsonized or non-opsonized bacteria and CHO cells expressing full length or tail less mutant CR3. The results show that serum-opsonized Schu S4 associates equivalently with full length CR3 and the tail less mutant CR3 expressing CHO cells whereas non-opsonized Schu S4 associates poorly (Fig. 8B). These results provide direct evidence for the key role of the CR3 cytoplasmic tail in mediating outside-in signaling immune suppression pathway (s) and for CR3/TLR2 cross talk in this process.

### *Lyn* kinase plays a critical role in CR3-mediated immune suppression during phagocytosis of Schu S4

Our results thus far identified a critical role for CR3 in mediating immune suppression during *F. tularensis* infection. The highly virulent Schu S4 exploits host defense mechanisms by using CR3 as the phagocytic receptor which suppresses host defense immune responses by inhibiting MAPK activation. However, the question still remained as to the signaling cascade downstream of CR3 that leads to MAPK inhibition.

A recent study [29] showed that CD11b can negatively regulate TLR signaling by activating Syk and promoting the degradation of MyD88 and TRIF. To explore this possibility, we examined the activation of Syk upon infection with serum-opsonized or non-opsonized Schu S4. Our results indicated that Syk is transiently activated upon Schu S4 infection, but there is no difference between serum-opsonized and non-opsonized Schu S4 infection at all time points studied (5, 15 and 30 min). Also MyD88 and TRIF protein levels following serum-opsonized and non-opsonized Schu S4 infection are not changed (data not shown). Thus, these data suggested that neither Syk nor Syk-mediated degradation of MyD88 and TRIF is involved in CR3-mediated immune suppression in human macrophages.

CR3 belongs to the β_2_ integrin family. Src family kinases (SFKs) Hck, Fgr and Lyn are known to control β_2_ integrin signal transduction in leukocytes [57]. Among these we were particularly interested in Lyn since it has been shown to play an important role in host defense. Lyn deletion led to impaired phagocytosis, elevated inflammatory cytokine production and increased apoptosis following *Pseudomonas aeruginosa* infection [58]. Lyn also negatively regulates TLR4 and TLR2 signaling in mouse macrophages and *in vivo*, which is partially mediated by the PI3K pathway [59].

**Figure 9:**
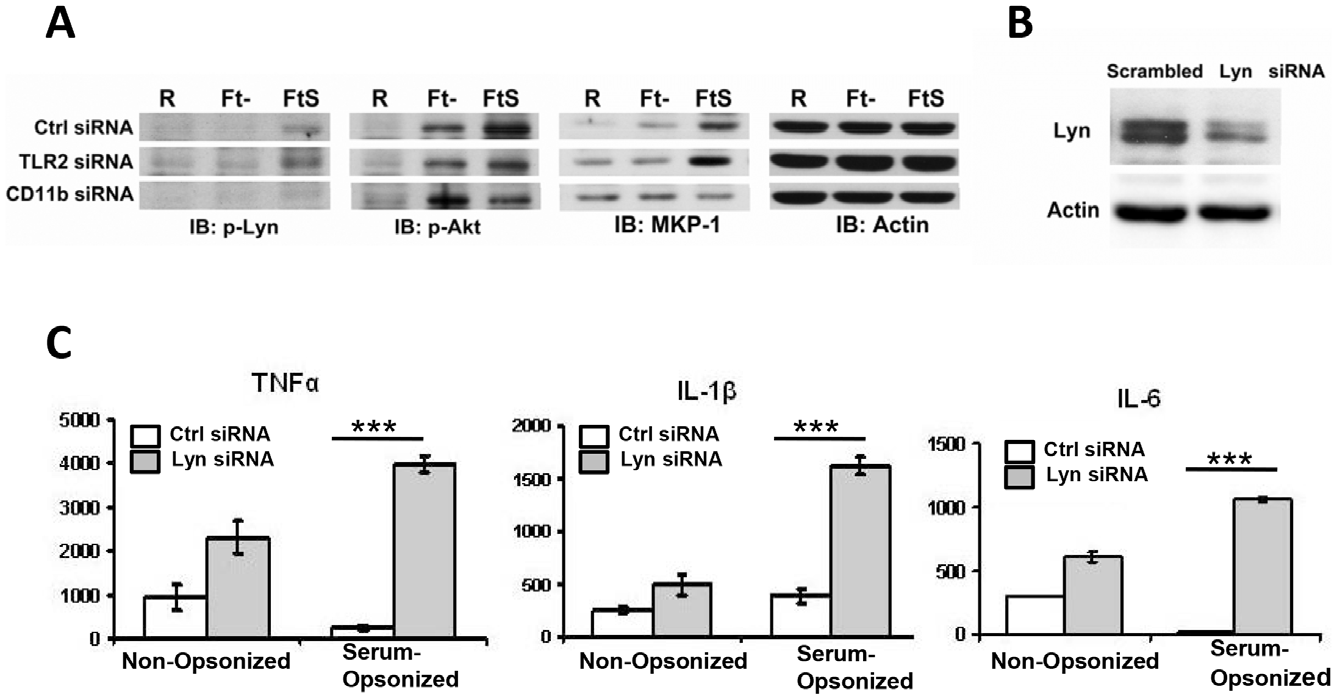
Lyn, AKT and MKP-1 are involved in CR3-mediated immune suppression during Schu S4 phagocytosis by macrophages. (A) hMDMs were transfected with control siRNA or siRNA targeting CD11b or TLR2. 48 h later cells were infected with non-opsonized or serum pre-opsonized *Ft* Schu S4 in the absence of serum for 30 min at MOI of 50. Infections were synchronized as described in the materials and methods. Cell lysates were subjected to SDS-PAGE and Western blot using antibodies against phospho-Lyn (Y396), phospho-Akt (T308), MKP-1 or β-actin. (B) Lyn siRNA knockdown in hMDMs. hMDMs were transfected with control siRNA or siRNAs targeting Lyn. 48 hrs later cell lysates were subjected Western blot using an antibody against Lyn. (C) 48 h after scrambled or Lyn siRNA transfection hMDMs were infected with Schu S4 in the presence or absence of serum at an MOI of 50∶1. 16 h post infection TNFα, IL-6 and IL-1β levels in culture supernatants were measured by ELISA. Data are representative of 3 independent experiments, each performed in triplicate. The data were analyzed by a two-tailed Student t-test *** p<0.001.

In order to answer the question whether Lyn is involved in CR3-mediated host immune suppression following Schu S4 infection, we first explored whether Lyn is activated downstream of CR3 and if so, whether its activation is affected by TLR2 activation which can activate CR3 through inside-out signaling. hMDMs were transfected with control siRNA, or siRNA targeting CD11b or TLR2, and then infected with either serum-opsonized or non-opsonized Schu S4. Lyn activation was measured by Western blot. As shown in Fig. 9A, in control siRNA transfected cells, Lyn was activated only when hMDMs were infected with serum pre-opsonized Schu S4 but not with non-opsonized Schu S4, consistent with the importance of C3 opsonization in Lyn activation. Moreover, when CR3 was knocked down, Lyn was no longer activated, whereas TLR2 knockdown had no effect. These results demonstrated that Lyn is activated downstream of CR3 but not TLR2.

In order to explore whether CR3-mediated host immune suppression is mediated by Lyn, we selectively knocked down Lyn by siRNA as described previously [60] (Fig. 9B). As shown in Fig S4, Lyn knockdown greatly impaired the uptake of Schu S4 by hMDMs, providing evidence that Lyn is required for CR3-mediated phagocytosis. The effect of Lyn on the host immune response was assessed by inflammatory cytokine production following Schu S4 infection. Compared with the limited immune response in control siRNA transfected cells, Lyn knockdown led to a significant increase in pro-inflammatory cytokine production, including TNFα, IL-6 and IL-1β, following serum-opsonized Schu S4 infection (Fig. 9C) despite the impaired uptake, providing evidence for a critical role of Lyn in CR3-mediated host immune suppression following Schu S4 infection.

### CR3 engagement leads to Akt activation and increased MKP-1 expression

The PI3K/Akt pathway has been shown to play a central role in the host immune response during *F. novicida* infection [18], including the production of TNFα [61]. In contrast, a recent study indicated that this pathway is involved in the immune suppression following LVS infection of mouse macrophages [62]. In support of a role of PI3K/Akt in *F. tularensis* immune modulation, Lyn has been shown to form a complex with PI3K/Akt in different cell types [63], [64] while TLR2 can also activate the PI3K/Akt pathway through Rac1 [65]. In order to examine whether the PI3K/Akt pathway is involved in CR3-mediated immune suppression and crosstalk with the TLR2 signaling pathway we infected control siRNA, CD11b siRNA or TLR2 siRNA-transfected hMDMs with serum pre-opsonized or non-opsonized Schu S4 and then examined the activation of this pathway by Western blot. As shown in Fig. 9A, 30 min after infection, the phospho-Akt level was consistently greater in hMDMs infected with serum-opsonized Schu S4, compared with hMDMs infected with non-opsonized bacteria, indicating that optimal engagement of CR3 at least transiently activates Akt. This was further supported by CD11b knockdown, which abolished the enhanced Akt activation seen in cells infected with serum pre-opsonized Schu S4 (below the level seen with non-opsonized bacteria). Besides CR3, TLR2 is also involved in Akt activation following Schu S4 infection (Fig. 9A). This was indicated by the increased level of phospho-Akt when cells are infected with non-opsonized bacteria which will engage CR3 in a limited fashion. Moreover, the increase seen following serum pre-opsonization was reduced in TLR2 siRNA transfected cells. As Akt is downstream of both pathways this could be an important point where the two pathways crosstalk.

MAPK activities are modulated through phosphorylation and dephosphorylation. MAPK phosphatases (MKPs) can dephosphorylate both phosphothreonine and phosphotyrosine residues thus serving as important negative regulators of MAPKs [66]. In order to examine if MKPs are involved in CR3-mediated immune suppression, we assessed MKP-1 levels during Schu S4 infection of hMDMs. Consistent with our observation of increased Akt and decreased MAPK levels upon infection with serum-opsonized Schu S4, the MKP-1 level also increased rapidly after serum pre-opsonized Schu S4 infection but not following infection with non-opsonized bacteria, suggesting a role for CR3 engagement. Consistent with this possibility, the increased MKP-1 level was abolished when CD11b was knocked down (Fig. 9A). In contrast, there was no effect following TLR2 knockdown. Together, these data map a pathway for CR3-mediated immune suppression that includes activation of Lyn and Akt, and an increased level of MKP-1.

## Discussion

*F. tularensis* is highly virulent with a very low dose of infection, which is often lethal before a fully effective adaptive immune response can be mounted [67], [68]. This high virulence and rapid lethality are at least partially due to the ability of *F. tularensis* to subvert or suppress host pro-inflammatory immune responses [16], [69]. *F. tularensis* infection of macrophages and dendritic cells leads to very limited secretion of the pro-inflammatory cytokines IL-1β, IL-6 and TNFα [13]–[15], [70] and unresponsiveness to subsequent stimulation by TLR agonists [14], [15]. In the current study, we provide evidence for a pathway that links CR3-mediated phagocytosis for *F. tularensis* with immune suppression that involves crosstalk with TLR2.

**Figure 10:**
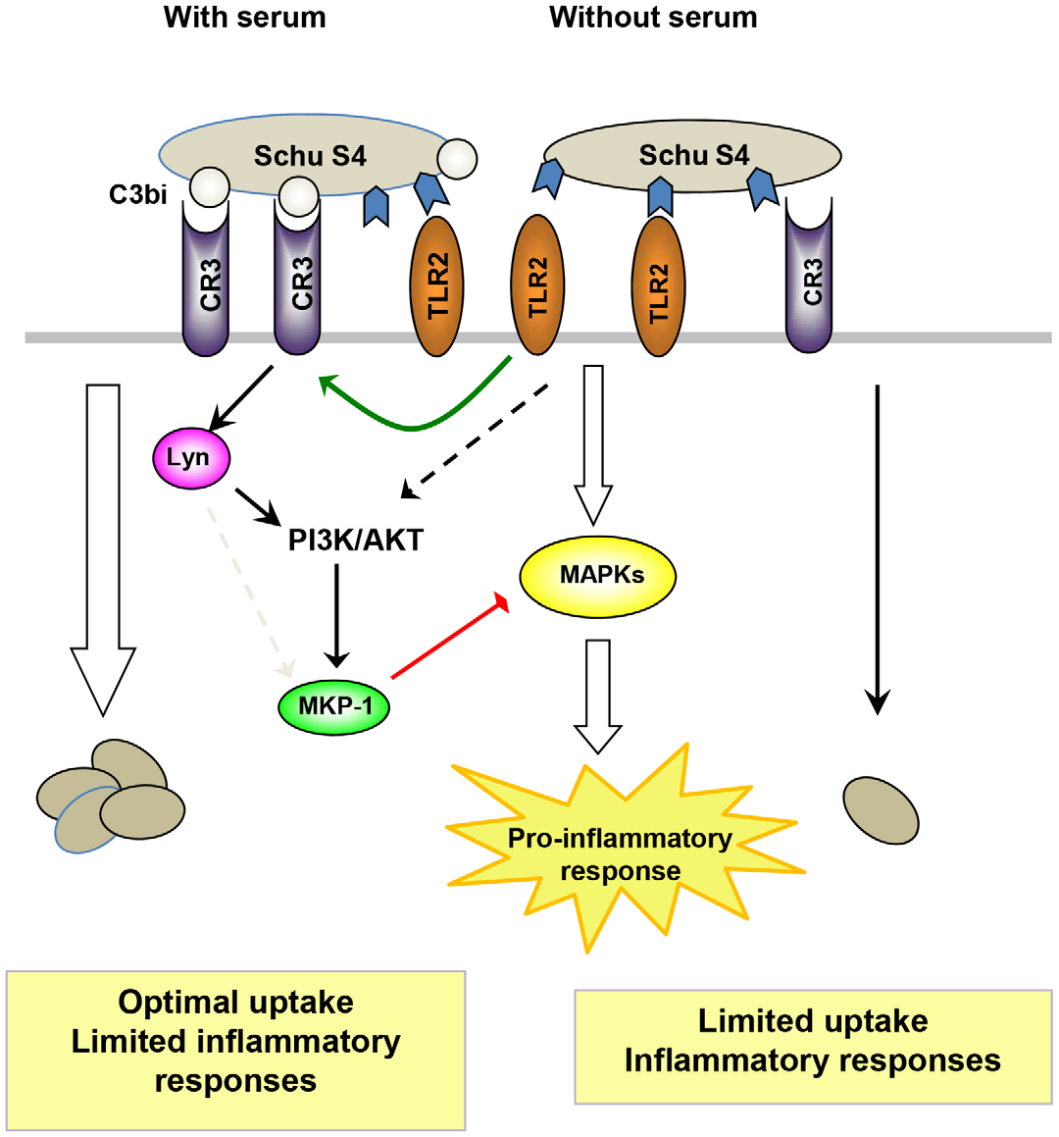
Model of CR3-mediated immune suppression and crosstalk with TLR2 signaling during the phagocytosis of Schu S4 by human macrophages. In the absence of serum, pathogen associated molecular determinants such as lipoproteins will be recognized by TLR2 which activates MAP kinase (ERK1/2 and p38) and NF-κB signaling pathways, and induces host pro-inflammatory responses. Phagocytosis is limited without the optimal engagement of CR3. In the presence of serum C3bi is deposited on the *Ft* Schu S4 surface, which optimally engages CR3 and enhances phagocytosis. At the same time, TLR2 is activated at least to some degree, leading to inside-out activation of CR3, which contributes to enhanced outside-in CR3 signaling and increased phagocytosis. CR3-mediated signaling activates Lyn and AKT, and leads to an increased MKP-1 level which results in inhibition of MAPK activity. This allows for increased phagocytosis simultaneously with a dampened host immune response.

Complement component C3 deposition on the *F. tularensis* surface has been previously characterized [25], [71], and optimal uptake of the highly virulent strain by human macrophages requires C3 deposition and its receptor CR3 [21]. Despite the fact that significantly more bacteria are phagocytosed by human macrophages in the presence of serum or C3, to our surprise we observed very limited pro-inflammatory cytokine production along with decreased activation of MAPK ERK1/2 and p38, and NF-κB under this condition. We excluded the possibilities that this C3-mediated immune suppression was due to a difference in the kinetics of phagocytosis or possible involvement of certain bacterial components/virulence factors, and confirmed the role of CR3 in this immune suppression via siRNA knockdown of the receptor. Further we provide direct evidence for the requirement of the CR3 cytoplasmic tail for C3 opsonized *F. tularensis*-mediated suppression of immune function in human macrophages. In addition we identify a pathway downstream of CR3 leading to immune suppression that includes Lyn, Akt and MKP-1. The engagement of CR3 leads to activation of Lyn, and the role of Lyn in immune suppression was confirmed by siRNA knockdown. CR3 engagement also led to transient activation of Akt and an increased level of MKP-1. These findings are consistent with the inhibition of ERK1/2 and p38 activation as well as limited pro-inflammatory cytokine production following Schu S4 infection. Thus, we identify a key pathway that links a receptor critical for *F. tularensis* phagocytosis with post-phagocytic signaling events important for immune suppression following Schu S4 infection, and the crosstalk downstream of two critical receptors in innate immunity: CR3 and TLR2 (Fig. 10).

The crosstalk revealed here is complex and involves interactions between TLR2 signaling and both inside-out and outside-in signaling of CR3 by *F. tularensis*. First, CR3 is required for efficient *F. tularensis* phagocytosis while TLR2 is also partially involved. This is likely through inside-out activation of CR3 which proceeds through Rac1, PI3K and cytohesin-1 [33], [34] and involves the actin cytoskeleton-binding protein talin (Fig. S3). The activated CR3 will then efficiently bind C3bi-coated bacteria, generate CR3 outside-in signaling involving Lyn and Akt, and negatively regulate TLR2-mediated immune responses by limiting the activation of MAPKs through increased MKP-1. Another possibility we cannot exclude from our studies is that the inhibition of TLR2 signaling happens at the level of receptor ligation. For example, it is possible that C3 deposition on *F. tularensis* can block the recognition of TLR2 ligands on the bacterial surface. In this regard, a recent study showed that *F. tularensis* grown in different media has distinct bacterial surface structures, which in certain cases shields TLR2 ligands on the bacterial surface and consequently blocks the host immune response [42]. The net result of CR3 engagement by *F. tularensis* is efficient phagocytosis and limited immune response. Although CR3 is present on the very cells that are meant to control *F. tularensis* infection, it allows for a relatively “silent” means of entry to macrophages.

We consistently observed a difference in the kinetics of ERK1/2 and p38 inhibition in our study. While the inhibition of ERK1/2 occurred as early as 5 min post infection, inhibition of p38 was not optimal until 30 min (Fig. 7B). This difference in kinetics has implications for MKP-1 regulation and function. In our study, the level of MKP-1 was not increased until 15 to 30 minutes post infection. In addition, although MKP-1 can deactivate ERK1/2 when it is highly induced, p38 is its preferred substrate [66]. Therefore taken together, although our current model of the signaling cascade can explain the inhibition of p38 activation, other mechanisms are likely to be involved in the early inhibition of ERK1/2. Further studies are underway to characterize the mechanisms enabling early ERK1/2 inhibition. Insight is provided by the modeling of C3-opsonized *F. tularensis* engagement of CR3 [72].

The observed CR3-mediated immune suppression is relatively specific to Schu S4. This is different from the immune suppression mediated by RipA, a *Francisella* protein that is conserved in all subspecies [52]. In our studies, *F. novicida* induced a much more robust immune response than Schu S4. In addition, serum or C3 had very little suppressive effect. *F. novicida* always has a much higher phagocytosis rate than Schu S4 in the absence and presence of serum (data not shown) suggesting the two strains use different means of cellular entry. Thus, differences in the early immune response to Schu S4 and *F. novicida* can be at least partly due to their engaging different receptors during phagocytosis. For example, it is possible that *F. novicida* more efficiently engages both CR3 and Fcγ receptors during phagocytosis, leading to a more robust immune response. Abundant natural antibody has been found on the surface of *F. novicida* that is capable of engaging Fcγ receptors [19]. Consistent with the notion that immune suppression is critical for the success of *F. tularensis* as a human pathogen, Schu S4 is highly virulent in humans whereas *F. novicida* is not. Beyond this, our studies do not rule out differences in bacterial surface-exposed ligands between Schu S4 and *F. novicida* that would still be present after PFA treating bacteria that could engage different receptors. We believe that differences in receptor-ligand interactions at the macrophage surface will lead to significant differences in the host cell response due to associated, unique, combinatorial signaling events.

The negative regulatory role of the PI3K/Akt pathway on pro-inflammatory immune responses has been demonstrated in other studies [73]–[75] including a recent study with LVS infection of mouse macrophages, which identified the role of Akt and MKP-1 in the immune suppression [62]. As LVS has a similar level of phagocytosis as Schu S4 (which is lower than that seen with *F. novicida*), and efficient uptake also requires serum-opsonization [19], it is possible that LVS uses a similar pathway as Schu S4 for immunosuppression. At a later stage of infection (24 h post infection), *F. novicida* activates while Schu S4 inhibits the PI3K/Akt pathway by differentially regulating SHIP-1 through induction of miR-155 [41]. Our studies support the idea that the induction of broad immune suppression by *F. tularensis* in the host occurs at multiple levels and by different mechanisms during the course of infection. The current study provides evidence that immune suppression mechanisms are initiated at the earliest state of infection, i.e. during the phagocytic process itself.

In addition to CR3, other complement receptors might also play a role during *F. tularensis* phagocytosis. C3bi can also be recognized by CR4 (CD11c/CD18) and CRIg [76]. These receptors might also be involved in the immune suppression observed during *F. tularensis* infection in the presence of serum. In addition, C1q is deposited on the *F. tularensis* surface (Clay CD & Schlesinger LS, manuscript in preparation). Interestingly, C1q suppresses LPS-induced pro-inflammatory cytokine production by NF-κB inhibition and CREB activation [77]–[79]. Opsono-phagocytosis of C1q-bound apoptotic cells occurs via CD91/calreticulin [80] and is immunosuppressive. Thus, it is intriguing to hypothesize that C1q receptors might also be involved in the immune suppression observed here. We are further exploring this possibility.

Signaling events associated with the complement system have been examined in the case of other pathogens. For example, *P. gingivalis* can cleave C5 to release C5a, which activates intracellular Ca^2+^ signaling and cAMP, leading to impaired iNOS-dependent killing in macrophages [81]. *P. gingivalis* can activate CR3 through inside-out activation downstream of TLR2 signaling, induces ERK1/2, and selectively down-regulates IL-12 expression [82], [83]. *Blastomyces dermatitidis* uses BAD1, a virulent factor on the pathogen surface to engage CR3 for TNFα suppression and immune evasion by unknown mechanisms [84]. In addition to its role in the phagocytosis of bacteria, CR3 is also responsible for clearance of C3bi-coated apoptotic cells without initiating vigorous immune responses [85]. In this context, CR3 ligation is immunosuppressive [86] and can inhibit IL-12 production [37], [87]. Although inside-out activation of CR3 through TLR2 has been reported for other pathogens [33], [34], [40], our data provide evidence that *F. tularensis* is able to take advantage of both inside-out and outside-in signaling of CR3 to achieve efficient entry as well as immune suppression for its own survival. Thus, we provide new information on how outside-in signaling of CR3 by a pathogen can negatively regulate TLR signaling. CR3 is a major receptor for intracellular pathogens such as *Mycobacteria* [88], *Leishmania* [89], [90] and *Legionella* [91]. Therefore it will be interesting to investigate whether these pathogens also employ similar signaling mechanisms related to CR3 in order to enhance their survival during infection. In this regard, during infection of murine macrophages with *Mycobacterium avium*, C3-depletion results in a significantly higher level of TNFα production [92]. TLR2, PI3K and cytohesin-1 mediated inside-out activation of CR3 has also been found to play a role in the phagocytosis of *Mycobacterium bovis* bacillus Calmette-Guerin (BCG) [34]. In the case of *Leishmania*, ligation of CR3 has been shown to inhibit the production of IL-12 [93].

During the course of our studies, we entertained the idea of extending them to the mouse model using CR3 knockout mice. We first examined the activation of ERK, p38 and Akt during serum-opsonized or non-opsonized Schu S4 synchronized phagocytosis by mouse bone marrow derived macrophages (BMDMs) from wild type C57/Bl mice. In stark contrast to the results obtained with human macrophages, the activation of ERK and p38 was significantly enhanced in mouse BMDMs incubated with serum-opsonized Schu S4 compared to non-opsonized Schu S4 (Fig. S5). This result indicates that the C3 opsonin-mediated inhibitory effect of Schu S4 on MAPKs is specific to human macrophages. In this regard, there is growing evidence that the mouse model does not recapitulate human disease for a number of infectious agents. In fact, both Schu S4 and *F. novicida* are highly virulent in mice whereas only the former is highly virulent in humans. Beyond this, we and others are beginning to see major differences between mice and man (and their cells), particularly in the innate immune system, where the array of PRRs on macrophages, for example, and their signaling cascades are quite different [94], [95].

In summary, our results demonstrate a pathway that links an important phagocytic receptor, CR3, on human macrophages with the down-regulation of host immune responses that allows for an efficient, but relatively “silent” mode of entry of the highly virulent *F. tularensis*. This involves active crosstalk between CR3- and TLR2-mediated signaling pathways. CR3-mediated immune suppression can be an important strategy used by *F. tularensis* for its success as a human pathogen. These results have broader implications in understanding the pathogenesis of other intracellular pathogens as well as the crosstalk between different PRRs and phagocytic receptors, especially TLRs and integrins.

## Materials and Methods

### Bacterial strains

*F. novicida* U112 (Fn) was obtained from ATCC and *F. tularensis* subspecies *tularensis* Schu S4 strain (Ft) was generously provided by Dr. Rick Lyons (Colorado State University, Fort Collins, CO). The bacteria were cultured on Chocolate II plates (Becton Dickinson, Sparks, MD). For some experiments, Schu S4 was passaged through THP-1 cells. Lysates from Schu S4-infected THP-1 cells were plated on Chocolate II plates and used in experiments in parallel with plate-grown only *F. tularensis*. Killed bacteria were prepared by fixation of bacteria with 2% PFA at room temperature for 10 min. They were then washed extensively with PBS and re-suspended in PBS. All work with the Type A Schu S4 strain was carried out in The Ohio State University BSL3 Select Agent facility in accordance with national and local approved BSL3 facility and safety plans.

### Reagents and antibodies

Human C3-depleted serum and human purified C3 were purchased from Comptech (Tyler, Texas). When needed, C3 was repleted at 1 mg/ml in C3-depleted serum.

Antibodies against phospho-p38, phospho-ERK1/2, phospho-Akt (T308), phospho- NF-κB p65, total NF-κB p65 and MyD88 were from Cell Signaling (Boston, MA). TLR2, MKP-1 (C-19) and β-actin antibodies were from Santa Cruz Biotechnology (Santa Cruz, CA). Antibody specific to phospho-Lyn (Y396) and Talin were from Abcam (Cambridge MA), and antibody against total Lyn was from Millipore (Billerica, MA). Mouse monoclonal antibody (H5A4) and rat monoclonal antibody (M1/70) against CD11b were obtained from the Developmental Studies Hybridoma Bank (The University of Iowa, Iowa City, IA). A monoclonal antibody against *F. novicida* LPS Fn8.2 was generated by Immuno-Precise Antibodies Ltd (Victoria, British Columbia, Canada) and a mouse monoclonal *F. tularensis* LPS antibody FB11 was from Abcam (Cambridge, MA).

Control scrambled small interfering RNA (siRNA), TLR2 siRNA [53], Lyn siRNA [60] and pre-designed siGenome smartpool targeting CD11b were purchased from Thermo Scientific Dharmacon RNAi Technologies (Lafayette, CO).

### Human monocyte-derived macrophage isolation and CHO cells

Human monocyte-derived macrophages (hMDMs) were isolated from human blood via venipuncture from healthy donors with no known exposure to *Francisella* following protocols approved by the Ohio State University Institutional Review Board. Written informed consent was provided by study participants and/or their legal guardians. Briefly, peripheral blood mononuclear cells (PBMCs) were isolated from heparinized blood as previously described [88]. PBMCs were then cultured in sterile screw-cap Teflon wells in RPMI 1640 plus L-glutamine with 20% autologous human serum at 37°C 5% CO_2_ for 5 days. PBMCs were then recovered from Teflon wells by chilling Teflon wells on ice and were subjected to siRNA transfection (see below), or re-suspended in RPMI with 10% autologous serum and allowed to attach in 12-well or 24-well tissue culture plates for 2–3 hrs at 37°C in 5% CO_2_. Lymphocytes were then washed away leaving MDM monolayers at a density of approximately 2.0×10^5^ cells/well for 24-well plates or 4.0×10^5^ cells/well for 12-well plates for *F. tularensis* infection.

CHO cells stably expressing full length CR3 and the tail less mutant form of CR3 as well as control cell lines (kind gifts of Dr. Douglas Golenbock, University of Massachusetts Medical School) were cultured and maintained in HAMs F12 medium at 37°C in 5% CO_2_. For infection, approximately 2.0×10^5^ cells/well for 24 well plates or 2.0×10^6^ cells/well for six well plates were used.

### Human serum preparation

Autologous sera were isolated from human blood obtained from healthy donors with no known exposure to *Francisella* by venipuncture. Sera were prepared as described before to preserve complement activity [19] and were aliquoted and stored at −80°C.

### hMDM siRNA transfection

Day 5 PBMCs were transfected with siGenome control siRNA or siRNAs targeting TLR2 (200 nM) [53], CD11b (400 nM) (Dharmacon predesigned smartpool siRNA) or Lyn (400 nM) [60]. These represent optimized concentrations based upon preliminary experiments where lower concentrations of siRNAs led to lesser effects on the target proteins and higher concentrations led to cell toxicity. Transfections were carried out using the Amaxa Nucleofector kit for human macrophages (Amaxa Biosystems, Gaithersburg, MD) as described earlier [53]. Briefly, 1×10^7^ day 5 PBMCs were re-suspended in 100 µl of human macrophages nucleofector transfection reagent, mixed with siRNAs, and incubated at room temperature for 5 min. Cells and siRNA mixtures were then nucleofected according to the manufacture's instruction. After transfection cells were immediately re-suspended in 1 ml of RPMI with 10% autologous serum and plated in 12-well tissue culture plate for Western Blotting and cytokine assays, or in 24-well tissue culture plates with acid-treated glass coverslips for confocal microscopy studies. After 2–3 hrs of attachment at 37°C with 5% CO_2_ hMDM monolayers were washed with warm RPMI, repleted with warm RPMI containing 20% autologous serum, and incubated at 37°C 5% CO_2_ for additional 48 hrs.

### CHO cell TLR2 transfection

The functional TLR2-expressing plasmid (YFP-TLR2) was transfected into CHO cells by using lipofectamine reagent (Invitrogen, Grand Island, NY, USA). Briefly CHO cells stably expressing full length CR3 or the tail less mutant form of CR3 were plated in 6 well (2.0×10^6^/well) or 24 well (2.0×10^5^/well) plates and incubated for overnight. The cells were washed, replenished with HAMs F12 media without serum, and the plasmid DNA lipofectamine complex was prepared according to the manufacturer's instructions and mixed with CHO cells. After 24 hrs of incubation cells were washed and replenished with HAMs F12 media with serum, incubated for 24 hrs and used for infections.

### *Francisella* infection of hMDMs and CHO cells: uptake and association assays

*F. novicida* or Schu S4 was cultured on Chocolate agar plates for 16 hrs and 40 hrs, respectively. Bacteria were re-suspended in PBS, and the multiplicity of infection (MOI) was approximated by measuring the optical density of the bacterial suspension at 600 nm, and confirmed by plating the inocula and counting CFUs. Bacteria were incubated with hMDM monolayers at an MOI of 50 in RHH (RPMI 1640 with L-glutamine, 10 mM HEPES and 0.25% human serum albumin) or RHS (RPMI 1640 with L-glutamine, 10 mM HEPES and serum) with 10% autologous serum, 10% C3-depleted serum (C3-dpl) or 10% C3-repleted serum (C3-rpl) for 15 min on a nutator for equal distribution, and then under stationary condition for another 45 min at 37°C, 5% CO_2_. Following infection, MDMs were washed with warm RPMI 1640 and incubated with 50 µg/ml gentamycin at 37°C 5%CO_2_ for 30 min to kill extracellular bacteria. MDM monolayers were then washed with PBS, lysed with 0.1% sodium deoxycholate, and *Francisella* uptake was numerated by serial dilution of the lysates and plated on chocolate agar plates for CFUs. MDMs were also fixed with 2% paraformaldehyde for 10 min at room temperature followed by an inside/outside differential staining protocol for attached and ingested bacteria as described below.

For CHO cell infections, transfected cells in incomplete HAMs F12 media were incubated with serum-opsonized or non-opsonized Schu S4 at an MOI of 50. Infections were synchronized by centrifuging at 4°C, 250×g for 10 min and incubated at 37°C, 5% CO_2_ for different time points. At each time point the cells were washed and lysed with TN-1 lysis buffer for Western blot or fixed with 2% paraformaldehyde for 10 min at room temperature followed by staining for Schu S4 for microscopy studies as described below.

### hMDM infection, lysis and Western blotting

*F. novicida* and Schu S4 were prepared as described above. Before infection, bacteria were pre-opsonized with 10% autologous serum, C3-depleted serum or C3-repleted serum at 37°C for 30 min, washed with ice-cold PBS and re-suspended in ice-cold PBS. A non-opsonized sample incubated with PBS instead of serum was also included in all experiments. MDM monolayers were washed with warm RPMI 1640, replenished with RHH and pre-chilled at 4°C for 10 min. Opsonized or non-opsonized bacteria were added to MDMs, and infections were synchronized by centrifuging at 4°C, 250×g for 10 min. hMDMs were then returned to 37°C 5% CO_2_ and incubated for the specified times. Cells were washed with PBS and lysed with TN-1 lysis buffer [50 mM Tris (pH 8.0), 10 mM EDTA, 10 mM Na4P2O7, 10 mM NaF, 1% Triton X-100, 125 mM NaCl, 10 mM Na3VO4, and 10 µg/ml each of aprotinin and leupeptin], incubated at 4°C for 5 min, and then centrifuged at 16,000× g at 4°C for 10 min to pellet the cell debris. Protein concentrations of the cleared cell lysates were measured using the Pierce BCA-protein assay kit (Thermo Scientific, Rockford, IL). Samples were subjected to separation by SDS-PAGE, and analyzed by Western blot with the different antibodies of interest. Protocols for CHO cell infections, cell lysis and Western blotting were the same.

### Cytokine ELISAs

MDMs were infected with *F. novicida* or Schu S4 without pre-opsonization in RHH or with RHS plus 10% autologous serum, C3-depleted serum or C3-repleted serum for 1 h as described above. Cell-free culture supernatants were collected at 16 hrs post infection. Cytokine concentrations (TNFα, IL-1β and IL-6) were measured by ELISA (R&D Systems, Minneapolis, MN) according to the manufacturer's instructions.

### Immunofluorescence microscopy

hMDMs were infected with *F. novicida* or *F. tularensis* Schu S4 at specified MOIs for different time points Monolayers were washed three times with PBS and fixed with 2% paraformaldehyde followed by an inside/outside differential staining protocol to differentiate extracellular and intracellular bacteria as previously described [50]. Briefly, after blocking with 5% goat serum and 0.5% BSA extracellular bacteria were detected with a monoclonal mouse anti-*Ft* LPS antibody FB11 (Abcam, Cambridge, MA) for 3 hrs at room temperature followed by an AF-488 conjugated goat anti-mouse secondary antibody. Cells were then permeabilized with 0.1% Triton X-100 in PBS at room temperature for 15 min, blocked with 5% goat serum and 0.5% BSA in PBS, incubated with the same primary antibody for 3 hr at room temperature followed by incubation with an AF-546 conjugated secondary antibody for 1 hr at room temperature. This allowed for the differential staining of extracellular (stained with both AF488 and AF546, shown as yellow or green) and intracellular (stained with only AF546, shown as red) bacteria. Host cell nuclei were stained with 0.05 µg/ml of 4′, 6′-diamidino-2-phenylindole (DAPI) at room temperature for 5 min.

The coverslips were mounted on glass slides and viewed using an Olympus FluoView 1000 confocal microscope. For quantification purposes, triplicate samples of at least 300 cells per group were counted on an Olympus BX51 epi-fluorescence microscope, and the results are presented as mean ± SD of representative data of at least three independent experiments.

For Talin co-localization experiments, hMDMs were infected with serum-opsonized or non-opsonized Schu S4 at MOI of 50 for 30 min at 37°C using the synchronized phagocytosis assay. Cells were washed three times with PBS and fixed with 2% paraformaldehyde followed by antibody staining for CD11b and Talin. Briefly, cells were then permeabilized with 0.1% Triton X-100 in PBS at room temperature for 15 min, blocked with 5% goat serum and 0.5% BSA in PBS, incubated with CD11b antibody for 3 hrs, washed 3 times, and incubated with Talin antibody for 3 hrs at room temperature. After washing, the cells were incubated with an anti-mouse AF488 (CD11b) and anti-rabbit AF594 (Talin) secondary antibodies for 1 hr at room temperature. The nuclei were stained with DAPI. For NF-κB p65 translocation studies in hMDMs, infected cells were fixed, permeabilized and blocked as described above, and incubated with NF-κB p65 antibody for 3 hrs at room temperature followed by anti-rabbit AF594 secondary antibody. The nuclei were stained with DAPI.

### Statistical analysis

Experiments were carried out independently at least 3 times with different donors. Results varied among donors but the patterns were the same with internal controls in each experiment. Representative results are shown. Prism software (GraphPad) was used to determine the statistical significance of differences in the means of experimental groups. An unpaired, two-tailed Student t-test was used when comparing the means from two groups of data and a 1-Way ANOVA and Tukey's Multiple Comparison Test was used when comparing three or more groups. The p value<0.05 were considered significant (*p<0.05, **p<0.005, and ***p<0.001).

## Supporting Information

Figure S1 **Host adaptation does not affect serum-mediated immune suppression by human macrophages.** Host adapted Schu S4 (Ft-T) was obtained by passaging bacteria through THP-1 cells. hMDMs were infected with Ft-T or non-passaged Ft Schu S4 at an MOI of 50:1 in RHH or RHS with 10% autologous serum. Extracellular bacteria were killed with 50 µg/ml gentamycin at 37°C for 30 min. Media was replenished and cell-free culture supernatants were collected at 16 hrs post infection. TNFα (A) and IL-1β (B) concentrations were measured by ELISAs. Uninfected resting cells (R) were included as a control. Data are representative of 3 independent experiments. The data were analyzed by a two-tailed Student t-test * p<0.05.
(TIF)

Figure S2 **CR3, partially activated by TLR2 inside-out signaling, is critical for Schu S4 phagocytosis by hMDMs.** hMDMs were transfected with scrambled siRNA or siRNAs targeting CD11b or TLR2. 48 h after siRNA transfection hMDMs were infected with non-opsonized or serum pre-opsonized *Ft* Schu S4 in the absence of serum for 5 min (A) or 30 min (B). Infected cells were subjected to differential staining as described in the Materials and Methods. Bacterial uptake was quantified as the number of bacteria that are inside or attached per cell. At least 300 cells were counted for every sample. Data are representative of 3 independent experiments performed in triplicate. The data were analyzed by a two-tailed Student t-test. * p<0.05, compared with control siRNA samples.
(TIF)

Figure S3 **Talin co-localizes with CD11b during phagocytosis of serum-opsonized** ***Ft*** **Schu S4 in human macrophages.** Human macrophages were infected with serum pre-opsonized or non-opsonized *Ft* for 30 min (synchronized phagocytosis). Cells were washed, fixed, permeabilized and incubated with CD11b (α chain of CR3) and Talin antibodies, washed, and further incubated with anti-mouse AF488 and anti-rabbit AF594 secondary antibodies. Slides were analyzed by confocal microscopy. The images in the left column show CD11b (green), middle column show Talin (red) and right column show the merged images of CR3 and Talin. The upper panel shows the cells infected with serum pre-opsonized *Ft* and lower panel those infected with non-opsonized *Ft*. Arrows indicate the co-localization of CR3 and Talin in macrophages infected with serum pre-opsonized *Ft*.
(TIF)

Figure S4 **Lyn is critical for Schu S4 phagocytosis by hMDMs.** hMDMs were transfected with scrambled siRNA or siRNA targeting Lyn. 48 h after siRNA transfection hMDMs were infected with serum pre-opsonized or non-opsonized *Ft* Schu S4 for 15 min. Infection was synchronized. Infected cells were subjected to differential staining as described in the Materials and Methods. Bacterial association was quantified as the number of bacteria that are inside or attached per cell. At least 300 cells were counted for every sample. Data are representative of 3 independent experiments performed in triplicate (mean ± SD).
(TIF)

Figure S5 **In contrast to human macrophages, engagement of the C3-CR3 pathway during Schu S4 phagocytosis increases the activation of MAPKs (ERK and p38) and Akt in mouse macrophages.** Bone marrow derived macrophages (BMDM) were infected with serum-opsonized Schu S4 (S) or non-opsonized Schu S4 (−) at an MOI of 50 by synchronized phagocytosis and incubated for different time points. Cell lysates were subjected to Western blot to measure activation of ERK (S5-A) p38 (S5-B) and Akt (S5-C) by using phosphor specific antibodies and then re-probed with total ERK, p38 and Akt antibodies. Shown is a representative Western blot from two independent experiments.
(TIF)
