# Correction: Fine Tuning Inflammation at the Front Door: Macrophage Complement Receptor 3-mediates Phagocytosis and Immune Suppression for *Francisella tularensis*

**DOI:** 10.1371/journal.ppat.1005504

**Published:** 2016-03-09

**Authors:** Shipan Dai, Murugesan V. S. Rajaram, Heather M. Curry, Rachel Leander, Larry S. Schlesinger

The authors would like to correct Figs 3 and 5, as errors were introduced in the preparation of these figures for publication. In Fig 3B, phosphorylation of JNK was cropped from a large experiment as shown in the uncropped blot in S1 Fig. The cropping for p-JNK and actin were performed incorrectly, shifted over to the right by one lane. The correct p-JNK and actin blots have been inserted into the corrected Fig 3. The raw blots for Fig 3B are presented below in S1 Fig. In Fig 5C, the authors determined that the wrong panel of the actin blot was inserted. To correct this error, the authors performed a new experiment exactly as performed in the original manuscript and present the results in the corrected Fig 5. The raw blots for Fig 5C are presented below in S2 Fig. The corrected version of Figs 3 and 5 can be viewed here.

**Fig 3:**
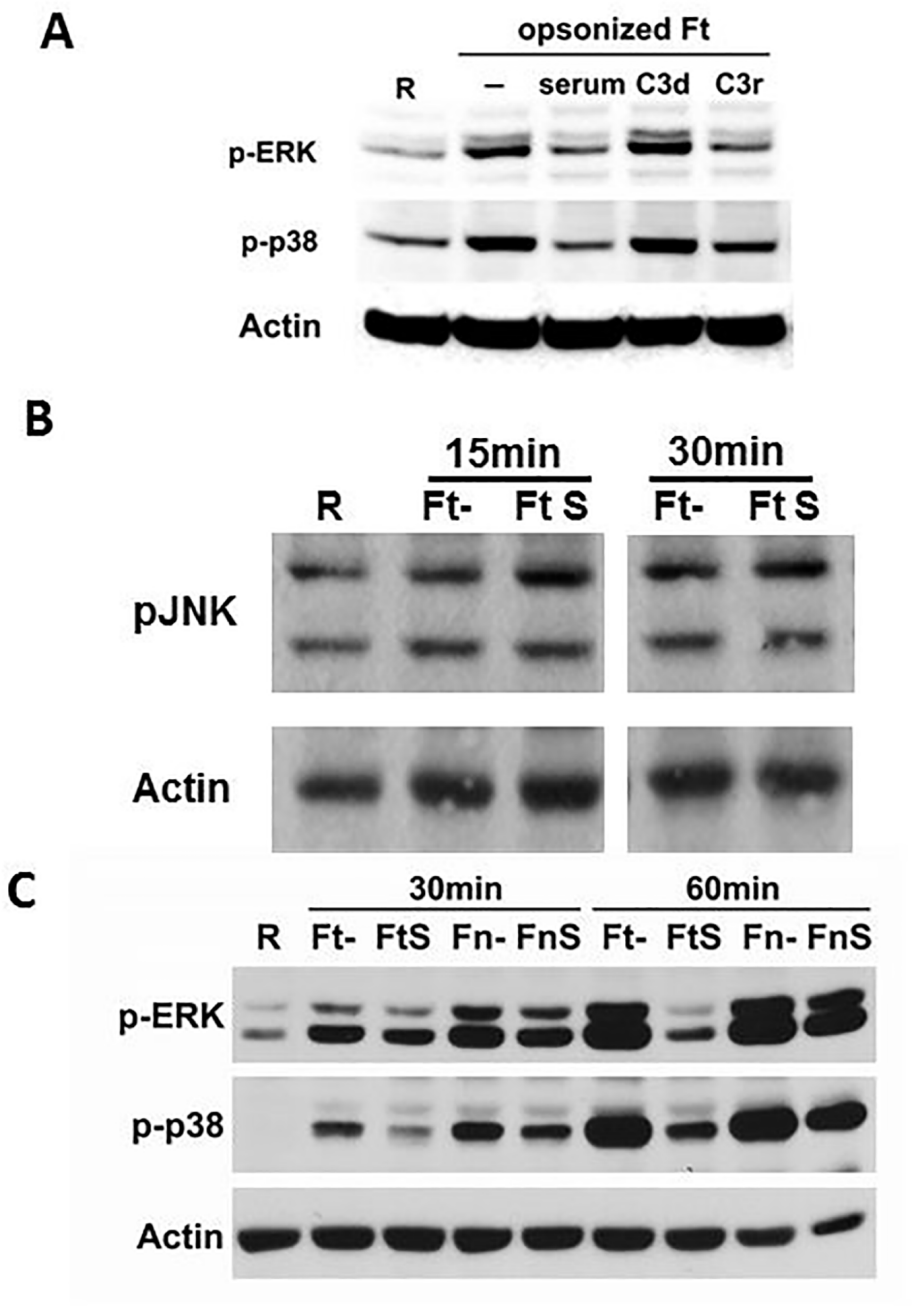
Serum components, specifically C3 opsonization, inhibit ERK1/2 and p38 activation during Schu S4 phagocytosis by macrophages. (A) Schu S4 was either non-opsonized, or pre-opsonized with 10% autologous serum, C3-depleted serum (C3d) or C3-repleted serum (C3r), and then used to infect hMDM monolayers at an MOI of 50:1 in RHH in the absence of serum. Infection was synchronized by centrifuging at 250xg for 10 min at 4°C, and incubated at 37°C for 30 min. (B) Schu S4 was either non-opsonized, or pre-opsonized with 10% autologous serum, and then used to infect hMDM monolayers at an MOI of 50:1 in RHH in the absence of serum. Infection was synchronized by centrifuging at 250xg for 10 min at 4°C, and incubated at 37°C for 15 or 30 min. (C) Schu S4 (Ft) or *F*. *novicida* (Fn) were either non-opsonized or serum pre-opsonized, and then used to infect hMDMs as in (A) for 30 or 60 min. MDM lysates were subjected to Western Blot. Uninfected resting cells (R) were included as control. Data are representative of 3 independent experiments.

**Fig 5:**
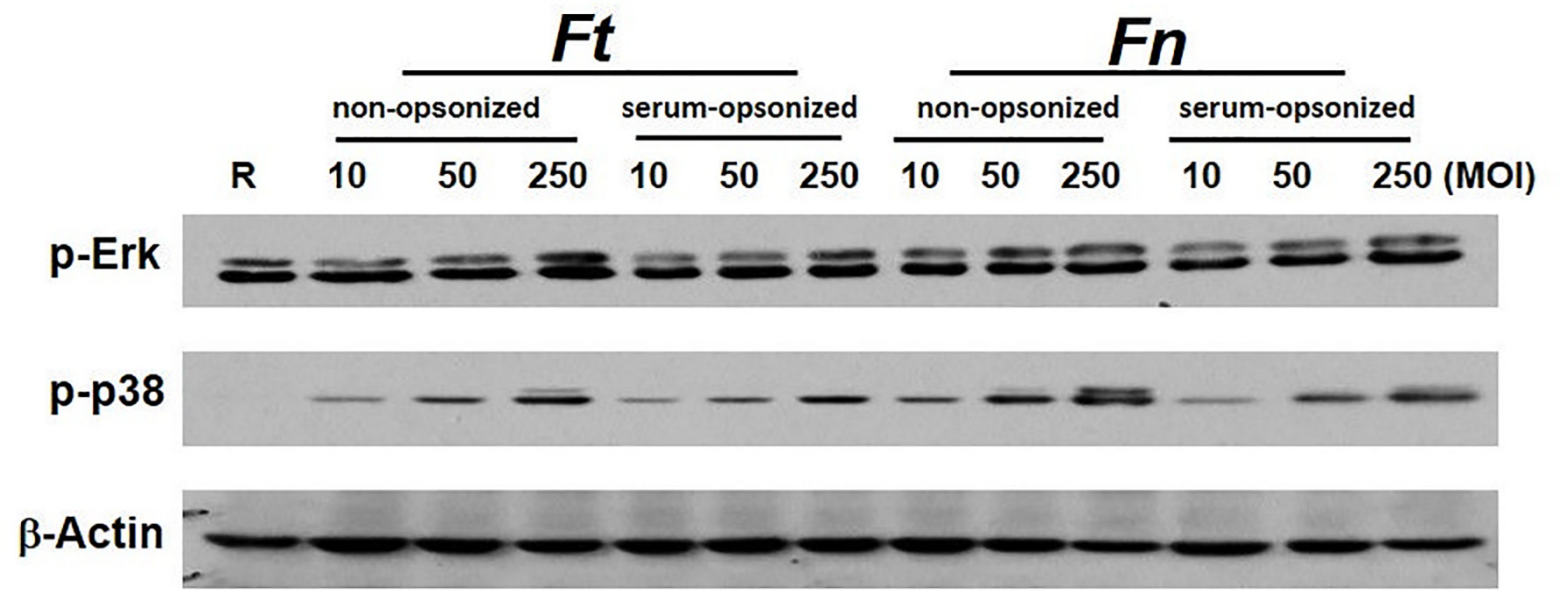
C3-mediated immune suppression is not due to a difference in the kinetics of Schu S4 phagocytosis. (A) In the presence of serum, there were more *Ft* Schu S4 attachment and phagocytosis. hMDMs were infected with non-opsonized or serum pre-opsonized *Ft* Schu S4 at an MOI of 50:1. Infections were synchronized by centrifugation at 4°C for 10 min at 250xg. At 5, 15 and 30 min post infection samples were washed extensively, fixed with 2% paraformaldehyde (PFA), and followed with inside/outside differential staining. The numbers of attached (extracellular) and phagocytosed (intracellular) bacteria were counted under an epi-fluorescence microscope. At least 300 cells were counted for every sample. Data are representative of 3 independent experiments performed in triplicate (mean ± SD). Non-opsonized vs. serum-opsonized, * p<0.05, ** p<0.005 Student *t*-test. (B) hMDMs were infected with Schu S4 (*Ft*) or *F*. *novicida* (*Fn*) at MOIs of 5, 20 and 100 in the presence (RHS with 10% autologous serum) or absence (RHH) of serum. Cell-free culture supernatants were collected after 16 h and cytokine levels were measured by ELISA. Data are representative of 3 independent experiments performed in triplicate (mean ± SD). (C) hMDMs were infected with non-opsonized or serum pre-opsonized Schu S4 (*Ft*) or *F*. *novicida* (*Fn*) at MOIs of 10, 50 or 250 for 30 min. Cell lysates were subjected to Western blot analysis. Data are representative for 3 independent experiments. (D) Schu S4 bacteria were either killed with paraformaldehyde or incubated with PBS (control) for 10 min at room temperature. hMDMs were then infected with non-opsonized or serum pre-opsonized live or PFA-killed Schu S4 at MOI of 50:1 for 30 min. Cell lysates were subjected to Western blot analysis. Data are representative of 3 independent experiments.

The authors confirm that these changes do not alter their findings. The authors have provided raw, uncropped blots as Supporting Information.

## Supporting Information

S1 Fig Raw data for Fig 3B. Schu S4 was either non-opsonized, or pre-opsonized with 10% autologous serum, C3-depleted serum (C3d) or C3-repleted serum (C3r), and then used to infect hMDM monolayers at an MOI of 50:1 in RHH in the absence of serum. Infection was synchronized by centrifuging at 250xg for 10 min at 4°C, and incubated at 37°C for the time points shown. MDM lysates were subjected to Western Blot with phosphor JNK antibody and reprobed with actin antibody.
(TIF)

S2 Fig Raw data for Fig 5C. hMDMs were infected with non-opsonized or serum pre-opsonized Schu S4 (*Ft*) or *F*. *novicida* (*Fn*) at MOIs of 10, 50 or 250 for 30 min. Cell lysates were subjected to Western blot analysis by using phosphor specific p38 and JNK antibodies, and reprobed with actin antibody.
(TIF)

## References

[1] Dai S, Rajaram MVS, Curry HM, Leander R, Schlesinger LS (2013) Fine Tuning Inflammation at the Front Door: Macrophage Complement Receptor 3-mediates Phagocytosis and Immune Suppression for *Francisella tularensis* . PLoS Pathog 9(1): e1003114 doi: 10.1371/journal.ppat.1003114 23359218 10.1371/journal.ppat.1003114 PMC3554622

