# Supplementary figures and images for: Fine Tuning Inflammation at the Front Door: Macrophage Complement Receptor 3-mediates Phagocytosis and Immune Suppression for Francisella tularensis
Source: PLoS Pathog. 2013 Jan 24;9(1):e1003114. doi: 10.1371/journal.ppat.1003114 (PMC3554622; doi:10.1371/journal.ppat.1003114)

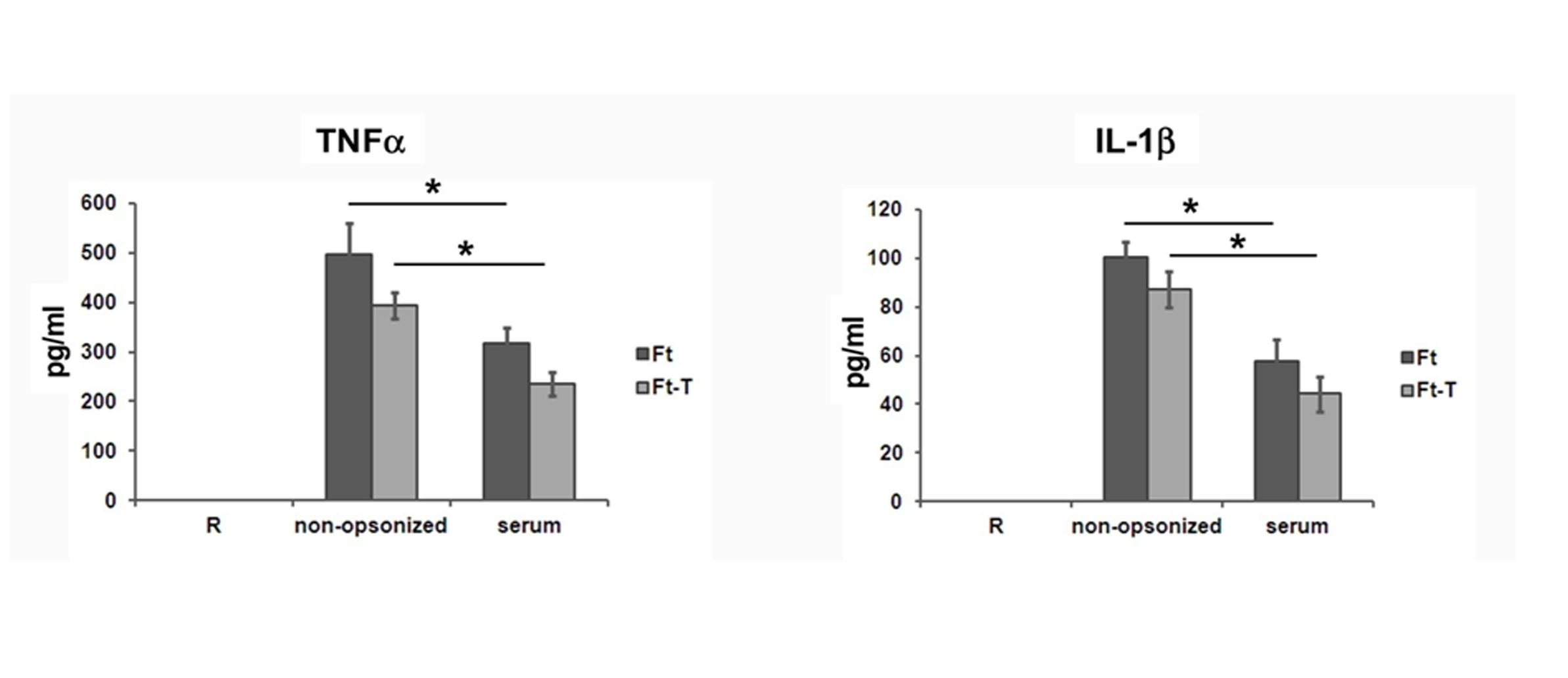

Supplement: Figure S1 — Host adaptation does not affect serum-mediated immune suppression by human macrophages. Host adapted Schu S4 (Ft-T) was obtained by passaging bacteria through THP-1 cells. hMDMs were infected with Ft-T or non-passaged Ft Schu S4 at an MOI of 50:1 in RHH or RHS with 10% autologous serum. Extracellular bacteria were killed with 50 µg/ml gentamycin at 37°C for 30 min. Media was replenished and cell-free culture supernatants were collected at 16 hrs post infection. TNFα (A) and IL-1β (B) concentrations were measured by ELISAs. Uninfected resting cells (R) were included as a control. Data are representative of 3 independent experiments. The data were analyzed by a two-tailed Student t-test * p<0.05. (TIF) [file ppat.1003114.s001.tif]

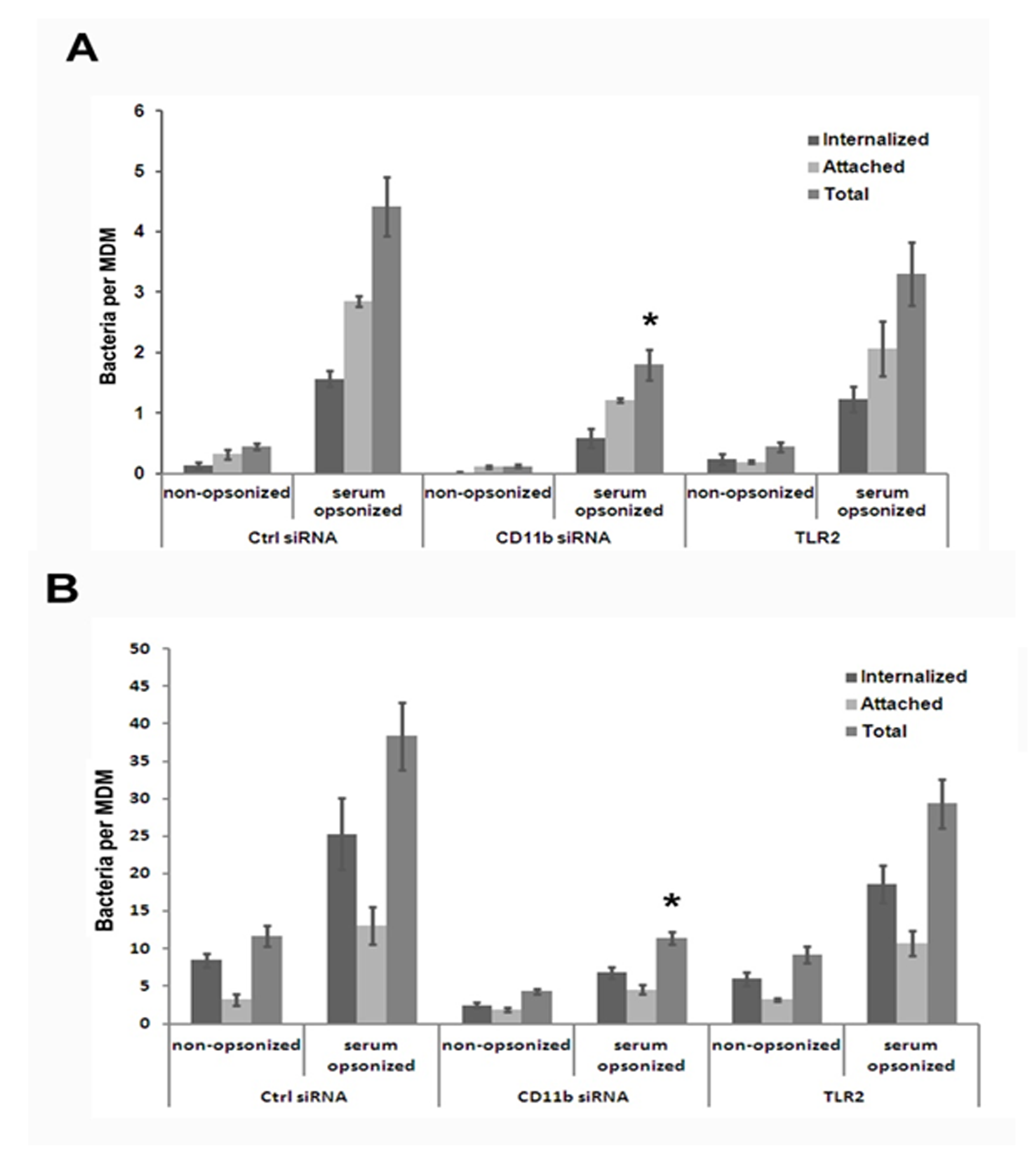

Supplement: Figure S2 — CR3, partially activated by TLR2 inside-out signaling, is critical for Schu S4 phagocytosis by hMDMs. hMDMs were transfected with scrambled siRNA or siRNAs targeting CD11b or TLR2. 48 h after siRNA transfection hMDMs were infected with non-opsonized or serum pre-opsonized Ft Schu S4 in the absence of serum for 5 min (A) or 30 min (B). Infected cells were subjected to differential staining as described in the Materials and Methods. Bacterial uptake was quantified as the number of bacteria that are inside or attached per cell. At least 300 cells were counted for every sample. Data are representative of 3 independent experiments performed in triplicate. The data were analyzed by a two-tailed Student t-test. * p<0.05, compared with control siRNA samples. (TIF) [file ppat.1003114.s002.tif]

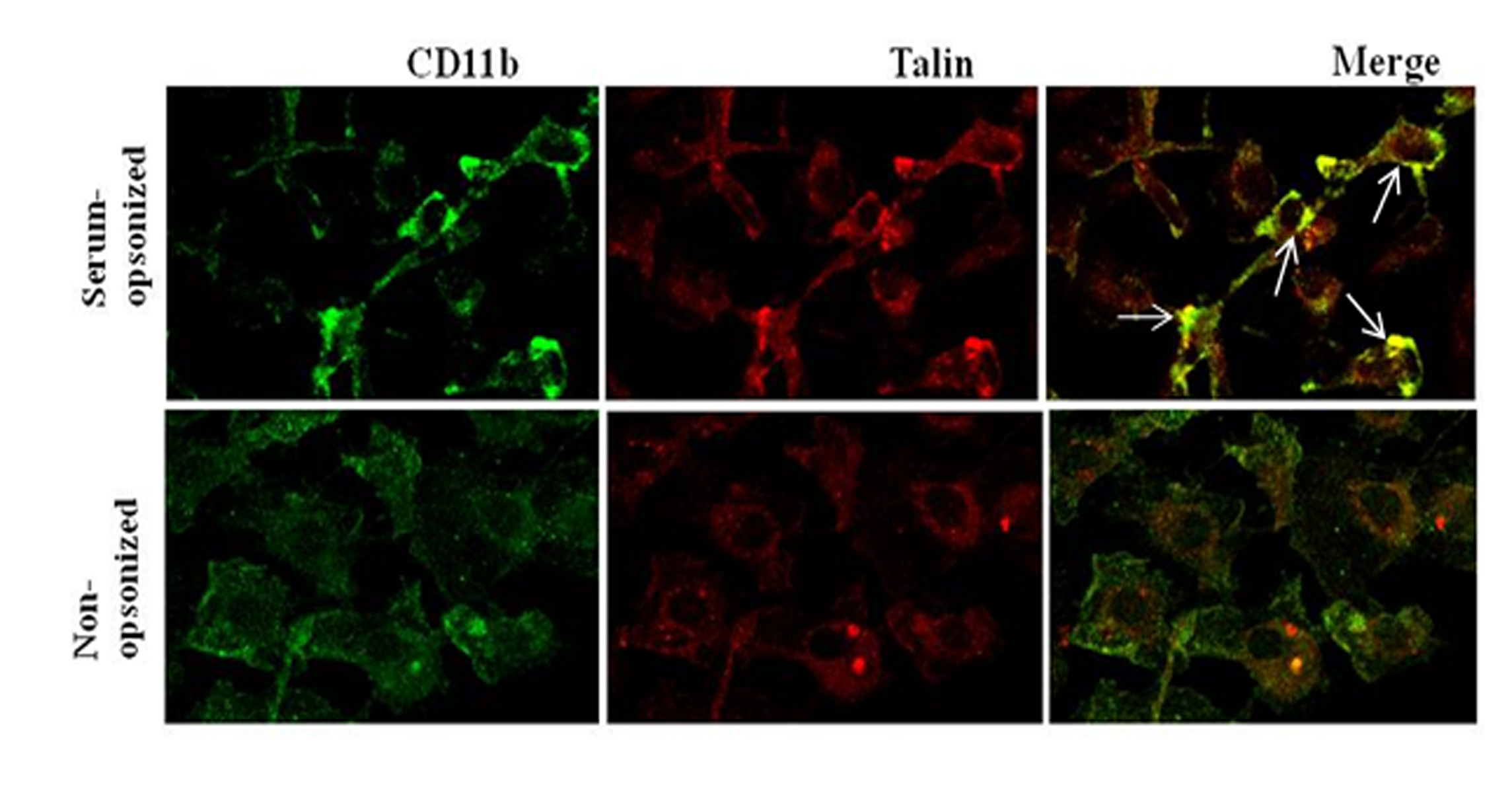

Supplement: Figure S3 — Talin co-localizes with CD11b during phagocytosis of serum-opsonized Ft Schu S4 in human macrophages. Human macrophages were infected with serum pre-opsonized or non-opsonized Ft for 30 min (synchronized phagocytosis). Cells were washed, fixed, permeabilized and incubated with CD11b (α chain of CR3) and Talin antibodies, washed, and further incubated with anti-mouse AF488 and anti-rabbit AF594 secondary antibodies. Slides were analyzed by confocal microscopy. The images in the left column show CD11b (green), middle column show Talin (red) and right column show the merged images of CR3 and Talin. The upper panel shows the cells infected with serum pre-opsonized Ft and lower panel those infected with non-opsonized Ft. Arrows indicate the co-localization of CR3 and Talin in macrophages infected with serum pre-opsonized Ft. (TIF) [file ppat.1003114.s003.tif]

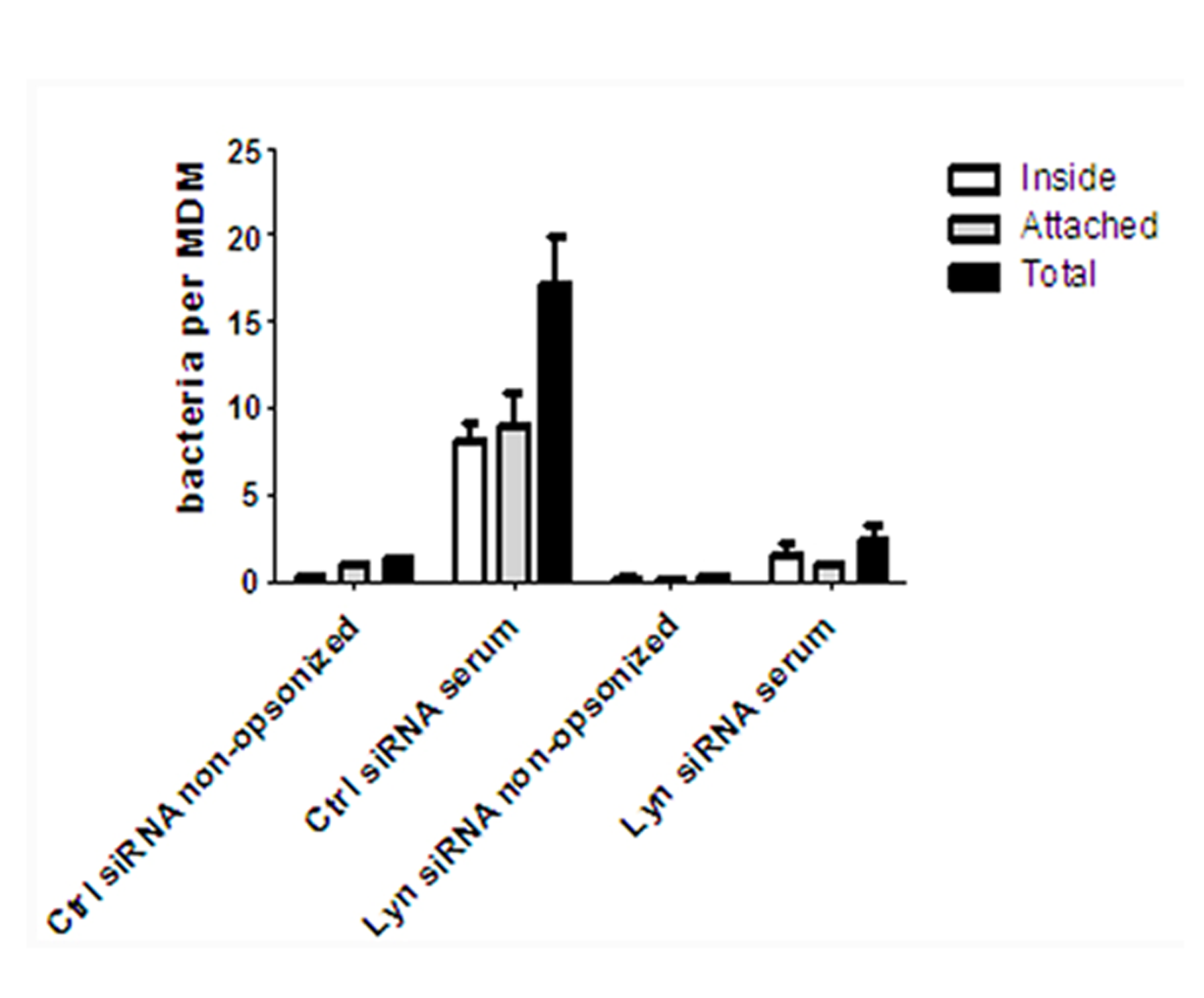

Supplement: Figure S4 — Lyn is critical for Schu S4 phagocytosis by hMDMs. hMDMs were transfected with scrambled siRNA or siRNA targeting Lyn. 48 h after siRNA transfection hMDMs were infected with serum pre-opsonized or non-opsonized Ft Schu S4 for 15 min. Infection was synchronized. Infected cells were subjected to differential staining as described in the Materials and Methods. Bacterial association was quantified as the number of bacteria that are inside or attached per cell. At least 300 cells were counted for every sample. Data are representative of 3 independent experiments performed in triplicate (mean ± SD). (TIF) [file ppat.1003114.s004.tif]

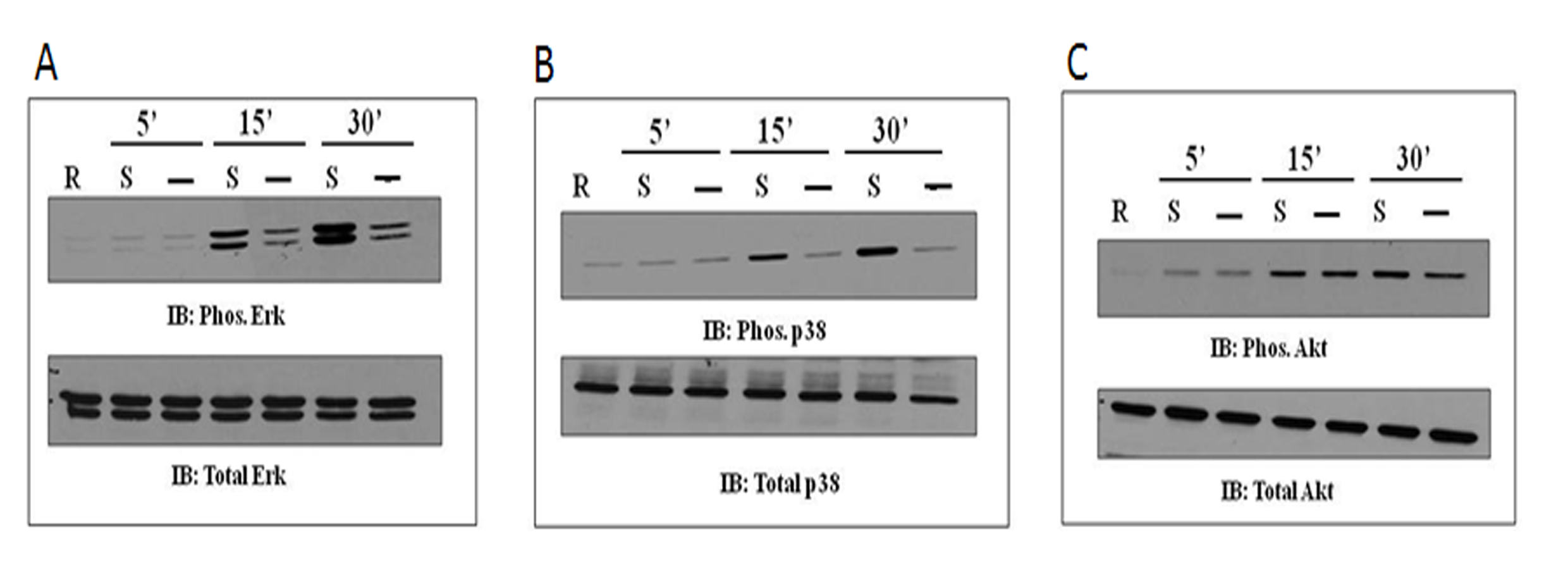

Supplement: Figure S5 — In contrast to human macrophages, engagement of the C3-CR3 pathway during Schu S4 phagocytosis increases the activation of MAPKs (ERK and p38) and Akt in mouse macrophages. Bone marrow derived macrophages (BMDM) were infected with serum-opsonized Schu S4 (S) or non-opsonized Schu S4 (−) at an MOI of 50 by synchronized phagocytosis and incubated for different time points. Cell lysates were subjected to Western blot to measure activation of ERK (S5-A) p38 (S5-B) and Akt (S5-C) by using phosphor specific antibodies and then re-probed with total ERK, p38 and Akt antibodies. Shown is a representative Western blot from two independent experiments. (TIF) [file ppat.1003114.s005.tif]
