# Supplementary figures and images for: Correction: Fine Tuning Inflammation at the Front Door: Macrophage Complement Receptor 3-mediates Phagocytosis and Immune Suppression for Francisella tularensis
Source: PLoS Pathog. 2016 Mar 9;12(3):e1005504. doi: 10.1371/journal.ppat.1005504 (PMC4784743; doi:10.1371/journal.ppat.1005504)

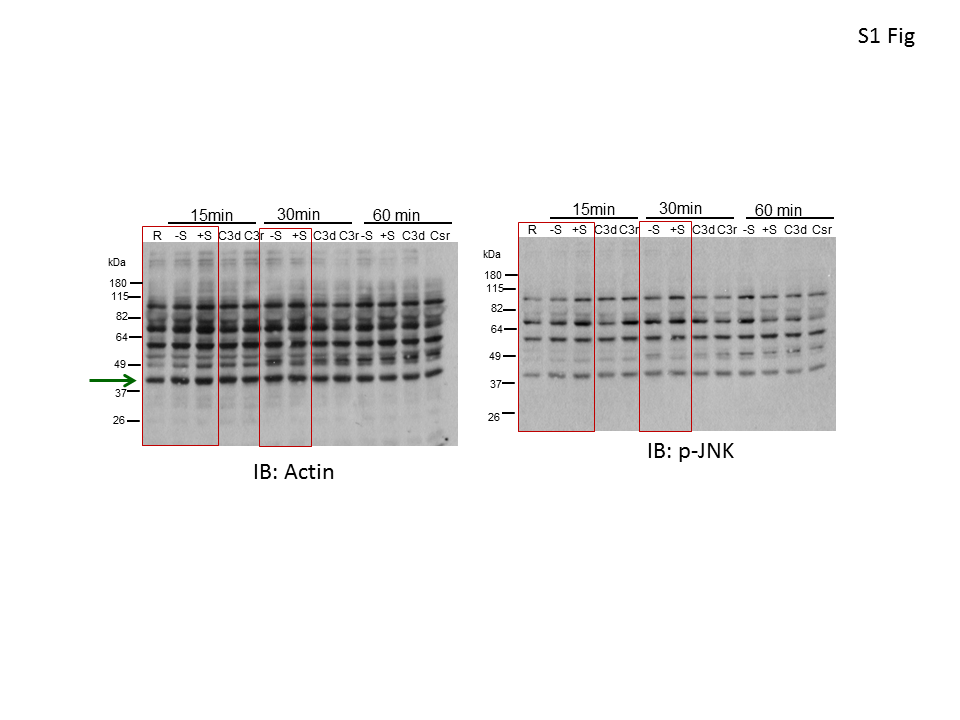

Supplement: S1 Fig — Schu S4 was either non-opsonized, or pre-opsonized with 10% autologous serum, C3-depleted serum (C3d) or C3-repleted serum (C3r), and then used to infect hMDM monolayers at an MOI of 50:1 in RHH in the absence of serum. Infection was synchronized by centrifuging at 250xg for 10 min at 4°C, and incubated at 37°C for the time points shown. MDM lysates were subjected to Western Blot with phosphor JNK antibody and reprobed with actin antibody. (TIF) [file ppat.1005504.s001.tif]

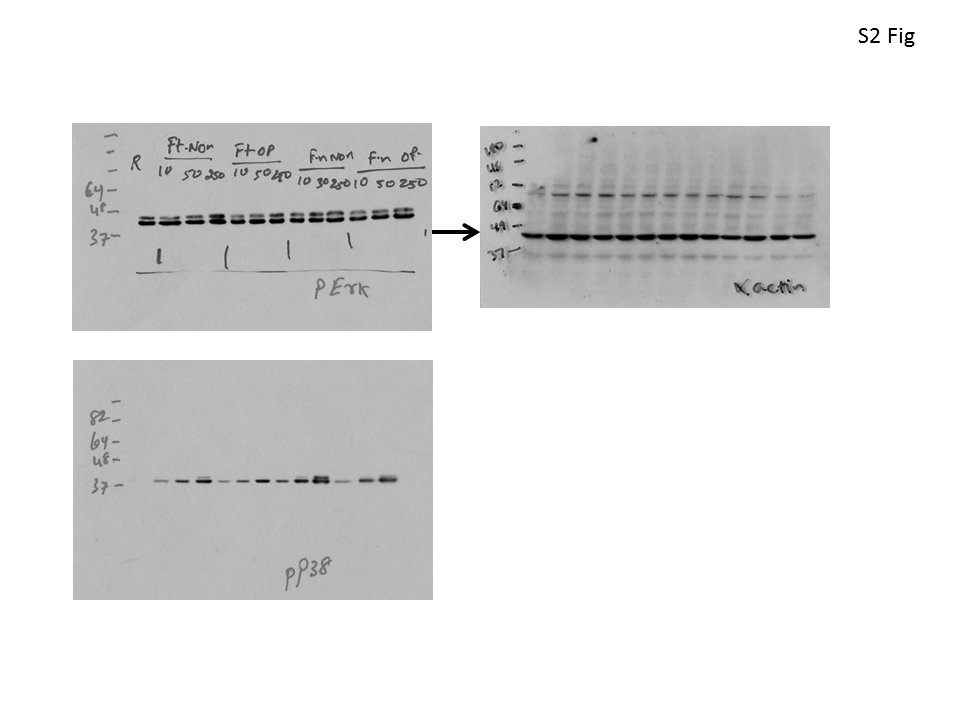

Supplement: S2 Fig — hMDMs were infected with non-opsonized or serum pre-opsonized Schu S4 (Ft) or F. novicida (Fn) at MOIs of 10, 50 or 250 for 30 min. Cell lysates were subjected to Western blot analysis by using phosphor specific p38 and JNK antibodies, and reprobed with actin antibody. (TIF) [file ppat.1005504.s002.tif]
